# Metabolic engineering reveals the relative importance of different sugar catabolic pathways during consumption of plant biomass by *Aspergillus niger*

**DOI:** 10.1016/j.crmicr.2025.100454

**Published:** 2025-08-05

**Authors:** Tania Chroumpi, Astrid Müller, Mao Peng, Mar Cubertorer Navarro, Robin Kuijpers, Agata Terebieniec, Jiajia Li, Lye Meng Markillie, Hugh D. Mitchell, Carrie D. Nicora, Chelsea M. Hutchinson, Vanessa Paurus, Samuel O. Purvine, Chaevien S. Clendinen, Galya Orr, Scott E. Baker, Miia R. Mäkelä, Ronald P. de Vries

**Affiliations:** a Fungal Physiology, Westerdijk Fungal Biodiversity Institute & Fungal Molecular Physiology, Utrecht University, Uppsalalaan 8, 3584 CT Utrecht, the Netherlands; b Environmental Molecular Science Laboratory, Pacific Northwest National Laboratory, 3335 Innovation Blvd, Richland, WA 99354, USA; c Department of Bioproducts and Biosystems, Aalto University, Kemistintie 1, 02150 Espoo, Finland

**Keywords:** Metabolic engineering, Sugar catabolic pathways, Plant biomass

## Abstract

To evaluate the impact of individual sugar catabolic pathways on the physiology of *A. niger* when using plant biomass as a carbon source, key pathways converting plant biomass derived monomers were blocked. The resulting deletion mutants were analyzed using wheat bran and sugar beet pulp as substrates. On both substrates, the strongest affected single-pathway mutants were the pentose catabolic pathway (PCP) (Δ*xkiA*) and glycolysis (Δ*hxkA*Δ*glkA*) deficient mutants. On wheat bran, which is rich in pentose sugars, blocking the PCP by deletion of *xkiA* strongly impacted both growth and gene expression. However, the effect was even stronger in Δ*hxkA*Δ*glkA* and similar to a strain in which all pathways were blocked, highlighting the crucial role of glycolysis and/or carbon catabolite repression in *A. niger* physiology. These results demonstrate the complexity of *A. niger* metabolism during growth on plant biomass and provide insights into aspects to consider during metabolic engineering to obtain fungal cell factories.

## Highlights

• *niger* uses multiple sugar catabolic pathways during growth on plant biomass.
• Blocking glycolysis reduces growth of *A. niger* on all tested carbon sources.
• *niger* can re-route metabolism when a specific sugar catabolic pathway is blocked.

## Graphical abstract

**Figure:**
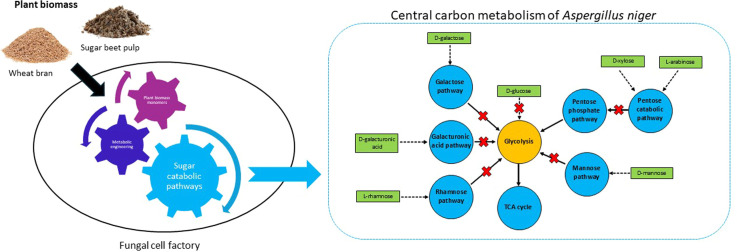

## 1 Introduction

Successful metabolic engineering strategies have been previously described in literature for the production of a great variety of primary metabolites of interest using filamentous fungi [reviewed by (Chroumpi et al., 2020b)]. However, it is important to understand how genomic information is translated into functional capabilities in order to enable more predictable redesign of fungal production platforms. Additionally, the large-scale production of these metabolites is usually hindered by various factors, such as low productivity and the high cost of the starting materials. In most studies, the engineered strains are mainly characterized, and their productivity is demonstrated using pure sugars as starting materials, which are rather expensive substrates. However, economically feasible biotechnological processes should ideally use low-cost feedstocks, e.g., plant biomass, and convert these directly into desired products.

The filamentous fungus *Aspergillus niger* is one of the most prominent fungal cell factories used by the biotechnology industry. It has been previously shown that metabolic engineering of *A. niger* can result in accumulation of valuable primary metabolites, such as xylitol, l-arabitol, lactic acid, citric acid, oxalic acid, l-galactonic acid, ascorbic acid, galactaric acid and itaconic acid (Chroumpi et al., 2020b; Dave and Punekar, 2015; de Jongh and Nielsen, 2008; Kobayashi et al., 2014; Kuivanen et al., 2012, 2015, 2016; Mojzita et al., 2010; Tong et al., 2019; Wiebe et al., 2010; Xie et al., 2020), but how metabolically engineered strains behave during growth on plant biomass has remained largely unknown. Filamentous fungi are highly versatile and efficient organisms in substrate utilization, especially with respect to plant biomass. During growth on plant biomass, multiple pentose (e.g., d-xylose, l-arabinose) and hexose (e.g., d-glucose, d-galactose, d-mannose, l-rhamnose) sugars become simultaneously available to the fungus. To utilize the highly varied sugar content of plant biomass, fungi possess a complex network of metabolic pathways to efficiently convert these sugars. Therefore, detailed understanding of central carbon metabolism is critical when applying metabolic engineering strategies in biotechnology.

**Fig. 1:**
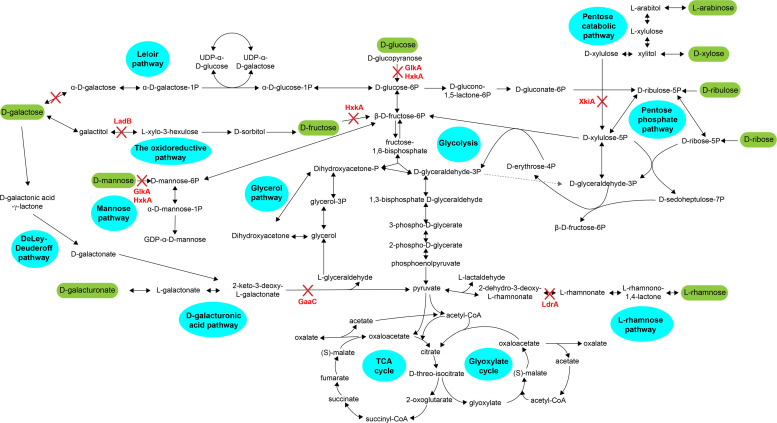
Schematic view of central carbon metabolism of *Aspergillus niger*. The compounds with green backgrounds are sugars commonly found in plant biomass in varying amounts. The pathways are indicated by a blue background. The enzymes encoded by the genes that are deleted in this study are highlighted in red font and the metabolic step that is blocked is indicate by a red cross. GlkA: glucokinase; HxkA: hexokinase; GaaC: 2-keto-3-deoxy-L-galactonate aldolase; XkiA: xylulokinase; LrdA: L-rhamnonate dehydratase; LadB: D-galactitol dehydrogenase; GalE: galactokinase.

The pathways involved in the catabolism of the most common plant biomass derived sugars (Fig. 1) have been studied in *A. niger* and most of the genes and enzymes involved in them have been identified and characterized. Conversion of d-glucose and d-fructose through glycolysis initiates with their phosphorylation by hexokinase (HxkA) and glucokinase (GlkA). The *Aspergillus nidulans* double deletion mutant Δ*hxkA1*Δ*glkA1* (Flipphi et al., 2003) was consequently unable to grow on these hexoses (Khosravi et al., 2018), while a similar mutant in *Aspergillus fumigatus* showed no growth on d-glucose, d-fructose and d-mannose (Fleck and Brock, 2010).

The pentose catabolic pathway (PCP) is responsible for the conversion of d-xylose and l-arabinose into d-xylulose-5-phosphate, which enters the pentose phosphate pathway (PPP) (Witteveen et al., 1989). Deletion of the *A. niger* xylulokinase encoding gene *xkiA* (Fig. 1) resulted in absence of growth on l-arabinose and d-xylose, while it was shown that for the other steps of the PCP are catalyzed by two or three enzymes and therefore blocking the pathway at these steps requires the deletion of multiple genes (Chroumpi et al., 2021a). While these PCP mutations can fully block growth of *A. niger* on pentoses, they are less critical for growth on more complex substrates, such as wheat bran (WB) and sugar beet pulp (SBP) (Chroumpi et al., 2021b). Additionally, production of xylitol using different PCP deficient *A. niger* mutants is highly depended on the carbon source used (Meng et al., 2022). The highest levels of xylitol were produced by Δ*xkiA* on d-xylose and by Δ*xdhA*Δ*ladA*Δ*sdhA* on WB in this study. A later study (Müller et al., 2025) demonstrated that further improvement can be achieved by constitutively activating the xylanolytic transcriptional activator XlnR (van Peij et al., 1998) together with deletion of the gene encoding the carbon catabolite repressor protein CreA (Dowzer and Kelly, 1989).

In the d-galacturonic acid catabolic pathway (GACP), d-galacturonic acid is converted to L‐galactonate, 2‐keto‐3‐deoxy‐L‐galactonate, and pyruvate and L‐glyceraldehyde, by enzymes encoded by *gaaA, gaaB*, and *gaaC*, respectively, while LarA (a.k.a. GaaD) converts L‐glyceraldehyde to glycerol (Alazi et al., 2017) (Fig. 1). Only deletion of *gaaC* resulted in complete growth arrest on d-galacturonic acid (Alazi et al., 2017), suggesting the presence of additional genes for the other metabolic steps in *A. niger*, similar as described above for the PCP.

Similarly, growth on l-rhamnose was only fully abolished after single deletion of *lrdA* (formerly known as *lraC*), while deletion of the upstream l-rhamnose catabolic pathway (RCP) genes *lraA* and *lrlA* (formerly known as *lraB*) only resulted in reduced growth on l-rhamnose (Khosravi et al., 2017), as did deletion of *lkaA*, involved in the last step of *A. niger* RCP (Chroumpi et al., 2020a).

In ascomycete filamentous fungi, d-galactose catabolism has been reported to consist of three pathways: the Leloir pathway (LP) (Frey, 1996; Katz and Rosenberger, 1970), the oxido-reductive d-galactose pathway (OGCP) (Fekete et al., 2004), and the De Ley-Douderoff pathway (Elshafei and Abdel-Fatah, 2001) (Fig. 1). In *A. niger*, deletion of the galactokinase encoding gene (*galE*), catalyzing the first step of the LP, abolished growth on d-galactose, while deletion of the d-galactitol dehydrogenase encoding gene (*ladB*) that is involved in the OGCP significantly reduced growth on d-galactose and abolished growth on d-galactitol (Chroumpi et al., 2022; Mojzita et al., 2012, 2011). No gene deletions and their phenotypes have been reported for putative genes of the De Ley-Douderoff pathway.

Despite these detailed studies into the pathways and their corresponding monosaccharides, the contribution of these catabolic pathways on the physiology of *A. niger* during growth on more complex substrates has not been studied. In this study, we aim to take the understanding of fungal central metabolism to a deeper level by dissecting the relative contribution of individual sugar catabolic pathways to the overall physiology of *A. niger* during growth on plant biomass in relation to the composition of the substrate and reveal to which extent the pathways can compensate for each other. To achieve this, we performed an in-depth analysis using selected metabolic mutant strains in which specific catabolic pathways are blocked and evaluated their phenotype as well as their transcriptomic response during growth on two plant biomass substrates, WB and SBP. Both substrates have a very low free sugar level, around 0.02 % in WB and 0.004 % in SBP (Peng et al., 2021), but differ significantly in their polysaccharide composition (Benoit et al., 2015). WB contains more l-arabinose, d-xylose and d-glucose, and consists mainly of cellulose and (arabino)xylan, while SBP is more abundant in l-arabinose, d-galacturonic acid, d-glucose and d-galactose, and contains cellulose, pectin and xyloglucan as its main polysaccharides (Table 1). In addition, by using a strain in which the common catabolic pathways for d-glucose, d-fructose, d-galactose, d-mannose, l-arabinose, d-xylose, l-rhamnose and d-galacturonic acid are all blocked, we aim to identify whether other components of plant biomass can support growth of *A. niger* when utilization of the major sugar components is blocked.

**Table 1:** Composition of wheat bran and sugar beet pulp (Benoit et al., 2015). Rha = *L*-rhamnose, Ara = *L*-arabinose, Xyl = *D*-xylose, Man = *D*-mannose, Gal = *D*-galactose, Glc = glucose, UA = uronic acid (D-glucuronic and/or D-galacturonic acid). The main polysaccharides have been extrapolated from the monosaccharide content. The monosaccharide content is given as g/100 g substrate. ND = not detected.

	Rha	Ara	Xyl	Man	Gal	Glc	UA	Starch	Main polysaccharides
Wheat bran	ND	14.4	21.4	0.1	0.7	28.5	ND	5.9	Cellulose, arabinoxylan
Sugar beet pulp	0.8	13.8	1.1	1.2	3.8	28.6	16.7	0.1	Cellulose, pectin, xyloglucan

## 2 Materials and methods

### 2.1 Strains, media and growth conditions

All *A. niger* strains used in this study are listed in Table 2. The uridine auxotrophic and non-homologous end-joining (NHEJ) deficient *A. niger* strain N593Δ*kusA* (reference strain) was used as parental strain for the construction of the single-pathway deletion mutants (Δ*xkiA*, Δ*gaaC*, Δ*galE*Δ*ladB*, Δ*lrdA* and Δ*hxkA*Δ*glkA*). All strains described in this study were deposited in the CBS strain collection of the Westerdijk Fungal Biodiversity Institute under accession numbers listed in Table 2. All strains were grown at 30 °C using Minimal Medium (MM, pH 6) or Complete Medium (CM, pH 6) with the appropriate carbon source (de Vries et al., 2004). For solid cultivation, 1.5 % (w/v) agar was added in the medium and, unless stated otherwise, all agar plates contained 1 % d-glucose as carbon source. As required, media of auxotrophic strains were supplemented with 1.22 g/L uridine, while a final concentration of 1.3 mg/mL of 5-fluoroorotic acid (5-FOA) was used for counterselection of strains carrying the *pyrG* marker gene on the self-replicating plasmid.

**Table 2:** Strains used in this study. PCP = pentose catabolic pathway, RCP = L-rhamnose catabolic pathway, GACP = D-galacturonic acid catabolic pathway, LP = Leloir pathway, OGCP = oxidoreductive D-galactose catabolic pathway.

Strain	Gene ID	Pathway blocked	CBS number	Reference
Reference	–	–	138,852	(Alazi et al., 2016)
Δ*xkiA*	NRRL3_04471	PCP	144,042	(Chroumpi et al., 2021a)
Δ*lrdA*	NRRL3_01495	RCP	144,313	(Chroumpi et al., 2020a)
Δ*gaaC*	NRRL3_05649	GACP	144,314	This study
Δ*galE*Δ*ladB*	NRRL3_06978 (*galE*) NRRL3_07283 (*ladB*)	LP OGCP	145,933	(Chroumpi et al., 2022)
Δ*hxkA*Δ*glkA*	RRL3_05100 (*hxkA*) NRRL3_03068 (*glkA*)	Glycolysis	146,917	This study
Δall (Δ*xkiA*Δ*galE*Δ*ladB* Δ*lrdA*Δ*gaaC*Δ*hxkA*Δ*glkA*)	See previous rows	PCP, RCP, GACP, LP, OGCP, Glycolysis	146,915	This study

Phenotypic analysis was performed in duplicates, on petri dishes (ø 6 cm) with vents containing MM agar supplemented with 25 mM monomeric, or 3 % (w/v) biomass substrates. Monosaccharide composition analysis of the biomass substrates, wheat bran (WB) and sugar beet pulp (SBP) used in this study is presented in Table 1 (Benoit et al., 2015). Spores were harvested from CM agar plates in ACES buffer, after five days of growth, and counted using a haemocytometer. Growth profiling plates were inoculated with 1000 spores in 2 μl and incubated at 30 °C for 5 days. As an exception, the d-galactose growth profiling plates were inoculated with mycelial plugs, grown between two hydrophilic polycarbonate membranes, and incubated at 30 °C for 14 days.

All liquid cultures were incubated in an orbital shaker at 250 rpm and 30 °C. For transfer experiments, the pre-cultures containing 250 ml CM with 2 % d-fructose in 1 L Erlenmeyer flasks were inoculated with 10^6^ spores/ml and incubated for 16 h. Thereafter, the mycelia were harvested by filtration on sterile cheesecloth, washed with MM and ∼0.5 g (dry weight) was transferred to 250 ml Erlenmeyer flasks containing 50 ml MM supplemented with 1 % WB or 1 % SBP. All cultures were performed in triplicate. After 8 and 24 h of incubation, the mycelia were harvested by vacuum filtration, dried between tissue paper and frozen in liquid nitrogen. All samples were stored at −80 °C until being processed.

### 2.2 Protoplast-mediated transformation, mutant purification and screening

For creation of all the mutants described in this study, the CRISPR/Cas9 system, as designed by (Song et al., 2018), was used. The Geneious R11 software (Kearse et al., 2012) was used for the identification of 20 bp guide sequences for our target genes against the *A. niger* NRRL3 genome (https://mycocosm.jgi.doe.gov/Aspni_NRRL3_1/Aspni_NRRL3_1.home.html). The guide sequences and plasmids used in this study are listed in Table S1A. To construct linear deletion DNA cassettes, the upstream and downstream flanking regions of *xkiA, galE, ladB, lrdA, gaaC, hxkA* and *glkA* were amplified by PCR using gene specific primers (Table S1B). PCR amplification was performed using Phusion™ High-Fidelity DNA Polymerase (Thermo Fisher Scientific), following manufacturer’s instructions, and using genomic DNA from reference strain as a template. The upstream reverse and the downstream forward primers were designed to harbor a barcode sequence [actgctaggattcgctatcg]. This sequence was used as the homologous region for the fusion of these two fragments in a PCR reaction, to generate the linear deletion DNA cassette. The amplified deletion cassettes were purified using the Illustra GFX PCR DNA and Gel Band Purification Kit (GE Healthcare Life Sciences). *A. niger* protoplasting was performed as previously described (Chroumpi et al., 2020a). Polyethylene glycol (PEG)-mediated transformation of *A. niger* protoplasts was performed as described in detail by (Kowalczyk et al., 2017). Transformations were carried out using 0.8 μg of ANEp8-Cas9-gRNA plasmid DNA together with 4–6 μg of purified linear deletion DNA cassette. All transformants, except for Δ*hxkA*Δ*glkA* and Δall, were plated on MM plates with 0.95 M sucrose. MM plates supplemented with 1.4 M d-xylose and 1 % (w/v) casein hydrolysate were used for plating the Δ*hxkA*Δ*glkA* and Δall transformants, respectively, since combined deletion of *hxkA* and *glkA* abolishes growth on sucrose. Five colonies per mutant were randomly selected from the transformation plates and streak-purified twice on MM plates supplemented with the appropriate carbon source. For *A. niger* colony PCR, genomic template DNA was isolated from mycelia of putative deletion strains using the Wizard® Genomic DNA Purification Kit (Promega). Correct mutants were identified by PCR amplification of the sequences flanking the CRISPR/Cas9 cut site, using primers listed in Table S1. Prior to storage, mutants were re-inoculated twice on MM plates supplemented with the appropriate carbon source and uridine, and subsequently on plates with 5-FOA (Nødvig et al., 2015) to remove the self-replicating plasmid.

### 2.3 High performance anion-exchange chromatography (HPAEC) analysis for profiling monosaccharides

The supernatants were analyzed by HPAEC-PAD on an ICS 5000 (Dionex) system equipped with a CarboPac PA1 column (2 mm ID x 250 mm) in combination with a CarboPac PA guard column (2 mm ID x 50 mm). Mobile phases were (A) 0.1 M NaOH and (B) 1 M NaOAc in 0.1 M NaOH. The column temperature was 20 °C and eluted with a linear gradient of B. Samples were diluted 5-fold and 1–200 μM of monosaccharides standard curves were used for identification and quantification in triplicates.

### 2.4 SDS-PAGE and enzyme activity assays

The culture supernatant of the reference strain and metabolic mutant strains grown in media containing 1 % WB or SBP were used to evaluate the produced extracellular CAZymes. 15 μL of culture supernatant was added to 5 μL loading buffer (10 % of 1 M Tris-HCL, pH 6.8; 42 % glycerol, 4 % (w/v) SDS; 0.02 % (w/v) bromophenol blue; 4 % of 14.7 M 2-mercapto-ethanol), incubated at 80 °C for 15 min, cooled on ice for 2 min and centrifuged at 10,000x*g* for 2 min. Finally, 15 μL were loaded onto 12 % (w/v) acrylamide SDS-PAGE gels with PageRuler Plus prestained marker (Thermo Scientific) and silver stained (Chevallet et al., 2006). All samples were evaluated in biological duplicates.

Extracellular enzyme activity was measured with 10 μL 0.1 % of substrates 4-nitrophenyl α-l-arabinofuranoside, 4-nitrophenyl *β*-d-glucopyranoside, 4-nitrophenyl *β*-d-xylopyranoside, and 4-nitrophenyl *β*-d-galactopyranoside. The reaction mixture also contained 50 μL 50 mM NaAc, pH 5.0 buffer, 10 μL supernatant sample, and 30 μL water. Samples were incubated for 30 min at 30 °C. The reaction was terminated with 100 μL of 0.25 M Na_2_CO_3_, and absorbance was measured at 405 nm using FLUOstar OPTIMA (BMG Labtech). All measurements were performed using both technical and biological triplicates. Differences in enzyme activities were determined using the Student’s two-tailed type *t*-test and significance was calculated as *p* < 0.05.

### 2.5 Transcriptome, proteome and metabolome data generation and analysis

The transcriptomic, proteomic and metabolic response of the reference strain and the deletion mutants induced after 8 and 24 h on 1 % WB or 1 % SBP was analyzed using protocols described previously (Chroumpi et al., 2021b).

Differentially expressed genes (DEGs) between mutants and corresponding reference strain were identified using the R package DESeq2 (Love et al., 2014), with all subsequent analysis performed in R unless otherwise stated. The list of plant biomass conversion genes (PBCs) were manually created from previous literatures, in which the gene function of sugar metabolic enzymes or CAZymes was predicted through ortholog mapping previously experimentally characterized enzymes (Li et al., 2022, 2023), and sugar transporters were identified by phylogenetic analysis of proteins containing PFAM domain PF00083 (Xu et al., 2024). A hypergeometric test was employed to determine whether the DEGs are significantly enriched the manually curated PBC gene set compared to the expected random occurrence within the *A. niger* genome. R packages, ggplot2 and Complexheatmap, were used for data visualization. Transcripts were considered differentially expressed if the DESeq2 fold change was > 2 or < 0.5 and Padj < 0.01. Principal component analysis and Pearson correlation were also analyzed based on the normalized counts from DESeq2.

## 3 Results and discussion

### 3.1 Phenotypic analysis on pure sugars revealed unexpected growth effects of the hexokinase/glucokinase mutant on non-related sugars

To selectively block the conversion of specific sugars we made a set of metabolic mutants in *A. niger*. Catabolism of d-glucose, d-mannose and d-fructose was blocked by combined deletion of *hxkA* (hexokinase) and *glkA* (glucokinase) (Khosravi et al., 2018), while pentose catabolism was blocked by deletion of *xkiA* (xylulokinase) (Chroumpi et al., 2021a). d-Galactose catabolism was blocked by combined deletion of *galE* (galactokinase) and *ladB* (galactitol dehydrogenase) (Chroumpi et al., 2022), l-rhamnose catabolism by deletion of *lrdA* (L-rhamnonate dehydratase) (Chroumpi et al., 2020a; Khosravi et al., 2017) and d-galacturonic acid catabolism by deletion of *gaaC* (2‐keto‐3‐deoxy‐L‐galactonate aldolase) (Alazi et al., 2017) (Fig. 1). To evaluate whether other components of the plant biomass substrates could still provide *A. niger* with sufficient energy to maintain growth on plant biomass, all these deletions were also combined in a single strain (Δall).

**Fig. 2:**
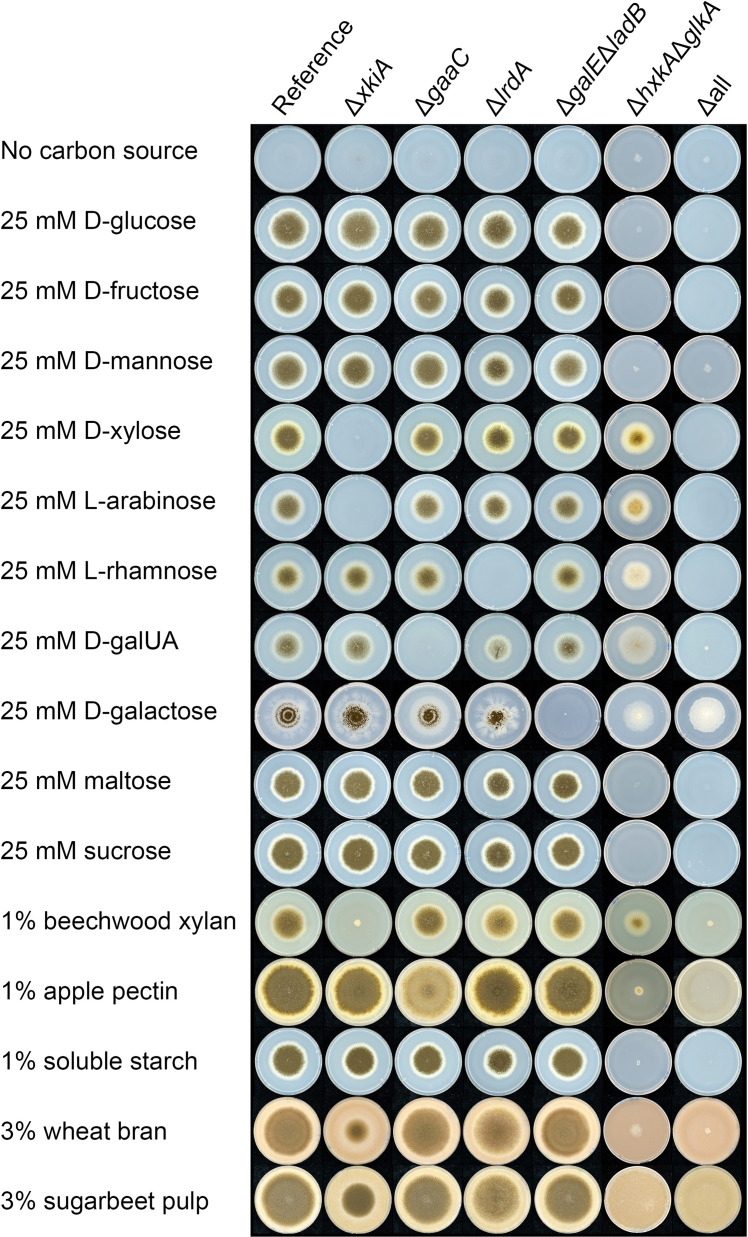
Growth profiles of the catabolic pathway mutants of *A. niger* on selected mono-, oligo- and polysaccharides and plant biomass substrates. Strains were grown for 5 days on MM plates with the carbon sources indicated in the figure in duplicate. Representative pictures are presented.

The phenotype of all the single-pathway deletion mutants (Δ*xkiA*, Δ*gaaC*, Δ*galE*Δ*ladB*, Δ*lrdA* and Δ*hxkA*Δ*glkA*) on their related sugars (Fig. 2) confirmed previously reported results (Alazi et al., 2017; Chroumpi et al., 2020a, 2022, 2021a; Khosravi et al., 2018). Single deletions of *xkiA, gaaC* and *lrdA* and combined deletion of *galE* and *ladB* resulted in abolished growth on pentoses, d-galacturonic acid, l-rhamnose and d-galactose, respectively, while their growth was not affected on the other monomeric sugar substrates. Growth of Δ*xkiA* was also abolished on xylan that mainly consists of d-xylose, while growth of Δ*gaaC* was significantly reduced on pectin that is particularly rich in d-galacturonic acid.

Double deletion of *hxkA* and *glkA* resulted in a much broader growth effect than was expected based on its role in the first step of glycolysis. As expected, no growth was observed on d-glucose, d-fructose and d-mannose, as well as oligosaccharides containing these sugars (Fig. 2). However, growth was also reduced on all the other tested substrates. While the substrate specificity of *A. niger* glucokinase is limited to d-glucose and d-mannose (Panneman et al., 1996), *A. niger* hexokinase can also phosphorylate d-fructose and d-glucosamine (Panneman et al., 1998). However, neither HxkA nor GlkA can phosphorylate d-galactose, l-sorbose, l-arabinose or d-xylose. HxkA is involved in the OGCP, catalyzing the phosphorylation of d-fructose to d-fructose-6P (Fekete et al., 2004), which could explain the reduced growth of Δ*hxkA*Δ*glkA* on d-galactose. However, the reduced growth of Δ*hxkA*Δ*glkA* on l-arabinose, d-xylose, l-rhamnose and d-galacturonic acid is likely due to an indirect effect.

Carbon catabolite repression mediated by CreA enables fungi to utilize the most favorable carbon source in the environment (Ruijter and Visser, 1997). This suggests that the reduced growth on these monosaccharides may be due to hexose accumulation causing a repressing effect on the expression of the genes of their related pathways. A role of CreA and accumulation of hexose in preventing the utilization of other monosaccharides is further supported by a study showing that glucose repression of several gene systems was fully retained in the *hxkA/glkA* mutant of *A. nidulans* (Flipphi et al., 2003).

Finally, the Δall strain that contains a combination of all the above-mentioned deletions could not grow on any of the tested substrates, except for d-galactose. This surprising finding suggests the possible induction of a backup pathway involved in d-galactose utilization, which is only activated after all the main *A. niger* sugar catabolic pathways have been blocked and which is not induced by d-galactose alone as there is no growth of Δ*galE*Δ*ladB* or Δall on d-galactose (Fig. 2). An explanation for this could be the suggested non-phosphorylated DeLey-Doudoroff pathway (Elshafei and Abdel-Fatah, 2001). While none of the six putative DeLey-Doudoroff pathway genes (Aguilar-Pontes et al., 2018) were significantly expressed or upregulated in the reference strain on d-galactose (Li et al., 2022), expression of all these genes except NRRL3_3123 was significantly increased during growth of both Δ*galE*Δ*ladB* and Δall mutants on SBP (Table S2). Analysis of the expression of these genes in Δall during growth on d-galactose will be required to confirm this.

### 3.2 Growth of *A. niger* on plant biomass substrates was mainly affected by deletion of *hxkA*/*glkA* and to a lesser extent by deletion of *xkiA*

Growth of Δ*gaaC*, Δ*lrdA* and Δ*galE*Δ*ladB* on WB and SBP was similar to that of the reference (Fig. 2) likely due to the high sugar heterogeneity of these substrates (Table 1) (Benoit et al., 2015) in combination with the high complexity and adaptability of fungal sugar metabolism that allowed for activation of alternative metabolic pathways to support growth. Deletion of *xkiA* resulted in significantly reduced growth compared to the reference strain on WB and SBP (Fig. 2). The residual growth is likely due to the production of additional enzymes with side-activity on the PCP sugars or re-routing of sugar catabolism towards utilization of other available plant derived monosaccharides (Chroumpi et al., 2021b). The better growth of Δ*xkiA* on SBP than WB could be attributed to the utilization of a wider range of alternative carbon sources in SBP, such as d-galacturonic acid. In a previous study (Kun et al., 2021), the low abundance of endoglucanases during growth on WB, combined with the slow growth on cellulose also indicated that cellulose is not a preferred carbon source for *A. niger*. This suggests that the residual growth of Δ*xkiA* on both biomass substrates cannot be explained by a switch to cellulose utilization but is more likely due to re-routing of pentose-derived metabolites.

The strongest reduction in growth on WB and SBP was observed for Δ*hxkA*Δ*glkA* and Δall (Fig. 2). This in agreement with the reduced growth and absence of growth of Δ*hxkA*Δ*glkA* and Δall, respectively, on all pure sugars. As both WB and SBP contain relatively low amounts of galactose (Table 1) this likely explains why this component did not result in significant growth on the plant biomass substrates. Although both substrates also contain other components (e.g., protein), apparently this was not able to rescue the growth of these two mutants. In contrast, growth of a strain in which all known genes encoding transcription factors involved in (hemi-)cellulose (XlnR, AraR, ClrA and ClrB) and starch (AmyR) utilization were deleted was better on wheat bran, which was suggested to be due to the utilization of the protein fraction of this substrate (Kun et al., 2021). This indicates that the impact of strain engineering at the metabolic level results in a stronger phenotype than regulatory level. This can also be explained by the effect of CreA as in both Δ*hxkA*Δ*glkA* and Δall higher extracellular d-glucose levels were observed throughout the cultivation (see 3.3), and involvement of CreA in the regulation of polysaccharide degradation during growth on plant biomass has been demonstrated (Peng et al., 2021). A previous study in *A. nidulans* in which a *hxkA/glkA* mutant strain was compared to a progeny in which *creA* was also mutated, demonstrated better growth on plant biomass substrates in the *creA* mutant (Khosravi et al., 2018).

### 3.3 The metabolic mutations specifically affect sugar release and consumption as well as enzyme production

Despite the very poor or no growth phenotype observed for Δall and Δ*hxkA*Δ*glkA* on plates, these strains did show growth in liquid cultures. We have demonstrated previously that *A. niger* shows different behavior in liquid and solid cultures (Garrigues et al., 2021; Kun et al., 2023) and these results confirm this. An explanation could be that in liquid culture *A. niger* is exposed to a constantly mixed substrate, while on plates it experiences carbon source gradients that result in a stronger growth effect. The growth observed in liquid cultures enables us to study the impact of the mutations in more detail at the transcriptomic, metabolic and enzymatic level.

To evaluate whether blocking the metabolic pathways affected the ability of the fungus to release and consume sugars originating from the biomass substrates, the monosaccharide levels in the culture filtrate and extracellular enzyme production were monitored over time in the cultures with WB or SBP as substrate.

**Fig. 3:**
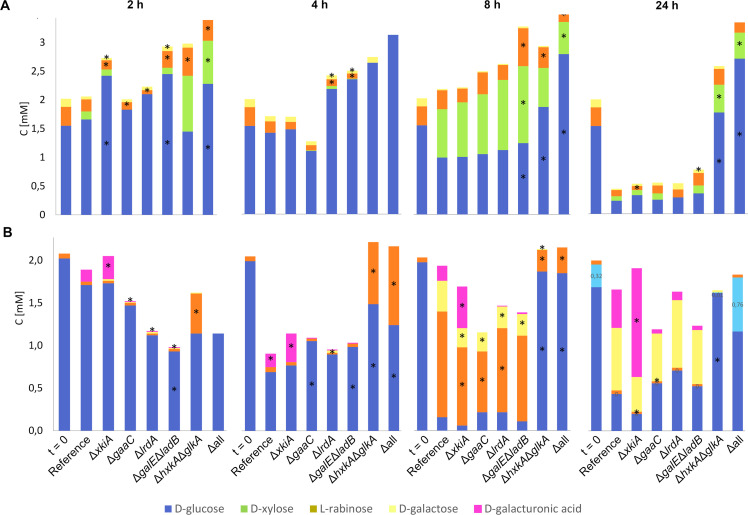
Sugar analysis of the culture filtrates of *A. niger* reference strain (CBS138852) and deletion strains on (A) 1 % wheat bran (WB) and (B) 1 % sugar beet pulp (SBP). *t* = 0 shows the WB and SBP composition before inoculation. Analysis was carried out using biological triplicates (graphs showing standard deviation for individual sugars are presented in Fig. S1). Statistical significance is represented by * (*p* < 0.05).

On WB, a reduction of the extracellular d-glucose concentration was apparent after 8 h of cultivation and even stronger after 24 h in all strains except Δ*hxkA*Δ*glkA* and Δall (Fig. 3A, Fig. S1A). This was accompanied by an increase in the extracellular d-xylose and l-arabinose concentration in all strains at 8 h that then reduced at 24 h, indicating both release and consumption of these sugars.

On SBP, a similar pattern was observed for d-glucose, but already at 4 h (Fig. 3B, Fig. S1B). In Δ*hxkA*Δ*glkA* an increase in free l-arabinose was already observed after 2 h and after 4 h for Δall, which reduced after 8 h and was nearly zero at 24 h, indicating release and consumption of this sugar, while no other sugars were observed in the culture filtrates of these strains. In the other strains, an increase in the concentrations of l-arabinose, d-galactose and d-galacturonic acid was observed after 8 h (Fig. 3B). After 24 h, the l-arabinose concentrations reduced to nearly zero, while the concentrations of the other two sugars increased, suggesting a broader range of sugar release and utilization in these strains compared to Δ*hxkA*Δ*glkA* and Δall.

The observed differences in sugar levels between the strains correlate with their differences in extracellular protein profiles (Fig. S2) and enzyme activity levels (Fig. S3), supporting the difference of Δ*hxkA*Δ*glkA* and Δall from the other mutant strains and the reference.

The depletion of the pentose sugars during growth of Δ*xkiA* on WB (Fig. 3), suggests the presence of enzymes with some kinase activity on the PCP intermediate d-xylulose or the conversion of PCP intermediates through alternative carbon catabolic pathways when they accumulate to sufficient levels. Interestingly, in most strains l-arabinose release and subsequent consumption occurs before that of d-galactose and d-galacturonic acid (Fig. 3), suggesting a sequential utilization of pectin-derived monosaccharides. It also correlates with high α-l-arabinofuranosidase activity already at 8 h (Fig. S3).

The inability of Δ*hxkA*Δ*glkA* to utilize hexoses (Fig. 2), resulted in prolonged accumulation of d-glucose in this mutant (Fig. 3) which supports the suggestion (see 3.2) that in this strain carbon catabolic repression mediated by CreA resulted in the repression of other metabolic pathways.

### 3.4 The transcriptome data support the differences in growth phenotype and extracellular sugar levels of the strains

**Fig. 4:**
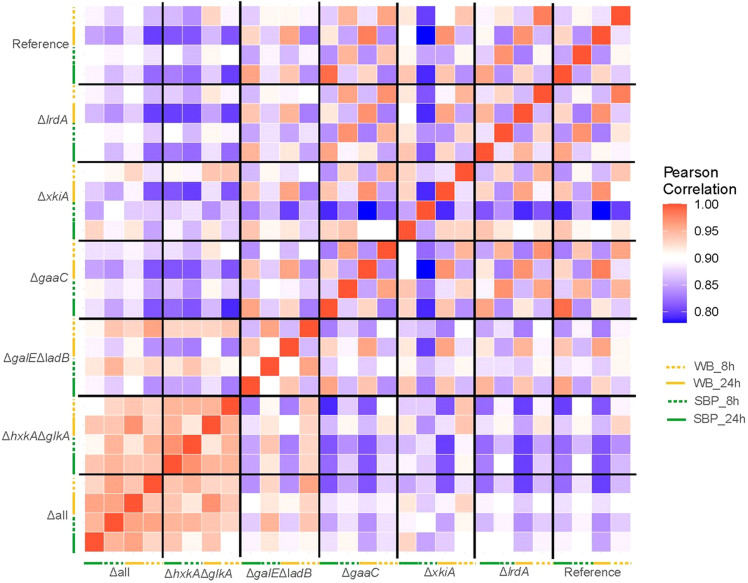
Similarity matrix of the transcriptome of all tested strains. The similarity between strains was determined by Pearson correlation coefficient based on average values of the normalized counts in each specific condition. WB=wheat bran, SBP=sugar beet pulp.

In support of the phenotypic and sugar level results (Fig. 2& 3), the correlation matrix of the transcriptome (Fig. 4) and the intra- and extracellular proteome and metabolome (Fig. S4) demonstrated clustering of the Δ*hxkA*Δ*glkA* and Δall strain, while the other strains are more similar to the reference.

**Fig. 5:**
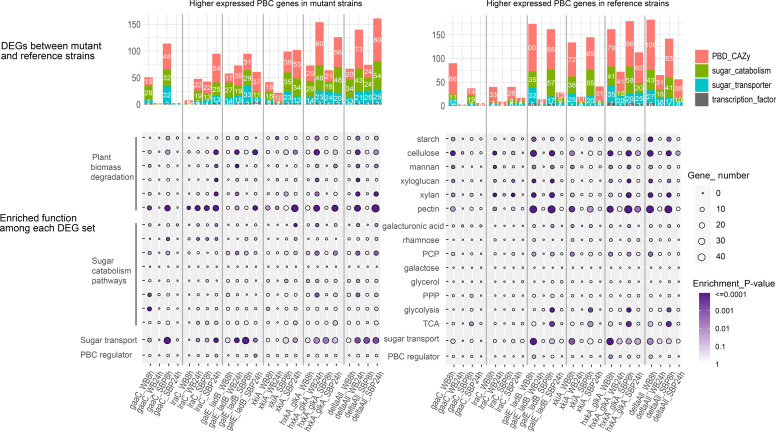
Enrichment barplot of up- and downregulated genes in the mutant strains compared to the reference. DEG = differentially expressed genes, PBC = plant biomass conversion, PBD = plant biomass degradation. The PBC genes were manually curated and divided into specific function categories, such as degradation of each specific polysaccharide and sugar metabolic pathway. The enrichment significance levels from low to high were indicate with colors from light to dark purple.

To analyze these effects in more detail, we focused specifically on genes related to plant biomass conversion (plant biomass degradation related CAZymes and transcription factors, sugar transporters, sugar catabolism). The transcriptome of this gene set showed a similar pattern as the whole transcriptome, with the highest number of differentially expressed genes (DEGs) compared to the reference strain was detected in Δ*hxkA*Δ*glkA* and Δall (Fig. 5). However, while there is an increase in up-regulated genes in both strains and on both substrates when the 8 h samples are compared to the 24 h samples, the opposite pattern is visible for down-regulated genes.

A clear time-dependent pattern was also observed for Δ*gaaC*, in which DEGs were detected on both substrates after 8 h, while after 24 h nearly no DEGs remained (Fig. 5). In the other strains a more diverse pattern is visible, with differences depending on substrate or time point, but also between up- or down-regulated genes (Fig. 5). This demonstrates the complex response of *A. niger* to crude plant biomass substrates when specific pathways have been blocked.

Heatmaps were generated for the expression of each of the four gene groups (CAZy, sugar transporter, metabolism, transcription factor) on the two substrates to show in more detail the similarity and differences of the mutant strains compared to the reference (Fig. S5).

On WB, the reference, Δ*gaaC* and Δ*lrdA* cluster together at 8 and 24 h, while Δ*xkiA* is often close to these strains (Fig. S5A-D). Δ*hxkA*Δ*glkA* and Δall cluster together at both time points, but at 8 h they also cluster with Δ*galE*Δ*ladB*, while this strain clusters with the reference, Δ*gaaC* and Δ*lrdA* at 24 h. The highest expressed CAZy and sugar transporter genes on WB in all strains are involved in (hemi-)cellulose degradation and hexose/pentose/disaccharide transport, respectively, and most of these are not affected by the metabolic mutations (Fig. S5A-B). In contrast, a broad set of metabolic genes is highly expressed in all strains (Fig. S5C), suggesting co-conversion of many of the biomass components.

On SBP, the reference, Δ*gaaC* and Δ*lrdA* cluster together at 8 and 24 h (Fig. S5E-H), similarly to what was observed on WB, but the other strains show a more diverse pattern. The Δ*xkiA* strain separates at 8 h from the other strains for the CAZy and metabolic genes but is similar to the reference and Δ*lrdA* for the transporters and to Δ*hxkA*Δ*glkA* and Δall for the transcription factors. Δ*hxkA*Δ*glkA* and Δall do not cluster together at both time points for all gene groups, unlike what was observed for WB (Fig. S5E-H), but at 8 h they mostly cluster together with each other and with Δ*galE*Δ*ladB*. The highest expressed CAZy and sugar transporter genes on SBP in all strains are involved in pectin and hemicellulose degradation. However, most of these are significantly reduced in Δ*hxkA*Δ*glkA*, Δ*galE*Δ*ladB* and Δall at 8 h (Fig. S5E) correlating with the lower presence of their inducing compounds (L-arabinose, d-galacturonic acid) (Fig. 3). Similar to what was observed on WB, a broad set of metabolic genes is highly expressed in both strains (Fig. S5G).

The similarity in the expression profile of the reference strain, Δ*gaaC* and Δ*lrdA*, suggests that blocking GACP and RCP by deleting *gaaC* and *lrdA*, respectively, do not significantly affect the physiology of *A. niger* on WB and SBP. This correlates with the similar growth of these strains on these substrates (Fig. 2). Up-regulation of several pectinolytic genes in Δ*gaaC* and Δ*lrdA* on both substrates (Fig. S5A & E) confirms the previously reported inducer accumulation in Δ*gaaC* (Alazi et al., 2017), but is contradictory to the previously suggested role of the metabolic product of LrdA as inducer (Khosravi et al., 2017).

The Δ*galE*Δ*ladB* mutant grown for 8 h on both WB and SBP also clustered together with Δ*hxkA*Δ*glkA* and Δall, while the expression of the sugar catabolic pathway genes in Δ*galE*Δ*ladB* at the later time point was more similar to the reference (Fig. S5C & G). However, the similarity of Δ*galE*Δ*ladB* and Δ*hxkA*Δ*glkA* at the transcriptional level at 8 h (Fig. 4) was not reflected in their growth profiles (Fig. 2). Therefore, the reasons behind the transcriptional similarity of these two strains on both substrates cannot be explained at this time, but requires further study. A possibility could be that *galE* and/or *ladB* are also involved in other pathways, and as a result their deletion significantly affected the transcriptional response of this mutant on WB and SBP.

Interestingly, an uncharacterized putative pentose/glycerol transporter (NRRL_4329) and sorbitol dehydrogenase (*sdhA*) are specifically and highly induced in Δ*galE*Δ*ladB* at 8 h, and in Δ*hxkA*Δ*glkA* and Δall at both time points on both substrates (Fig. S5B, C, F, G). Despite *sdhA* being assigned to the OGCP (Koivistoinen et al., 2012), a previous study already questioned this role as deletion of this gene did not affect growth on d-galactose or d-galactitol (Chroumpi et al., 2022). Our results also do not support this role as the expression pattern of *sdhA* is significantly different from that of *ladB* and *xhrA*, two other genes of the OGCP (Fig. S5C & G).

### 3.5 Blocking specific pathways results in diverse responses of genes belonging to other pathways

**Fig. 6:**
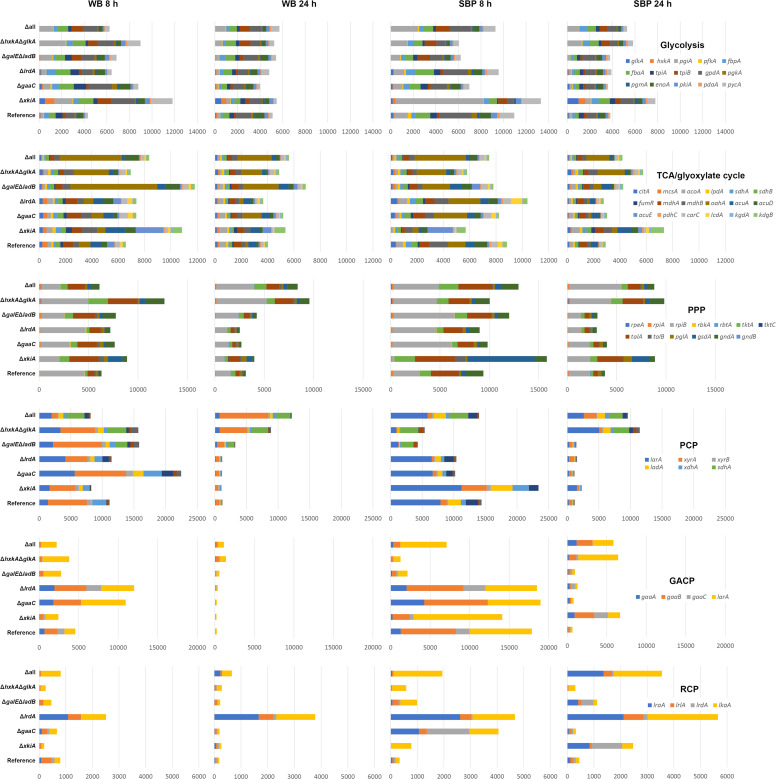
Comparative expression profiles of the cumulative expression levels of genes related to the same pathway. The pathways and pathway genes are indicated on the right. PPP = pentose phosphate pathway, PCP = pentose catabolic pathway, GACP = *D*-galacturonic acid catabolic pathway, RCP = *L*-rhamnose catabolic pathway.

To take a closer look at the influence of the metabolic mutations on the other metabolic pathways, the expression values of the genes of each pathway were cumulatively plotted (Fig. 6). This demonstrated that the expression of the genes of the core metabolic pathways (glycolysis, PPP, TCA/glyoxylate cycle) are only moderately affected in the metabolic mutants. In contrast, genes of the PCP are strongly reduced at 24 h on both substrates in all strains except Δ*hxkA*Δ*glkA* and Δall. A possible explanation for this is that the inducers of the related transcriptional activators XlnR and AraR, d-xylose and l-arabitol, respectively (Battaglia et al., 2011), are no longer sufficiently available in these strains, while they still are available in Δ*hxkA*Δ*glkA* and Δall. This does not fully match the extracellular sugar profiles (Fig. 3), but induction of gene expression is likely due to intracellular levels and can already be sufficient at very low levels (de Vries et al., 1999).

Expression of most genes of the GACP and RCP was reduced in Δ*hxkA*Δ*glkA* and Δall at 8 h, but possibly due to CreA mediated expression as discussed above that prevents induction of these pathway genes. At 24 h these genes have increased expression in these strains compared to the reference, suggesting that CreA-mediated repression mainly occurred at the early time points. In contrast, high expression of the GACP and RCP genes (Fig. 6) and genes encoding pectin-specific CAZymes (Suppl. Fig. 5E) was observed on SBP in Δ*xkiA*, which correlates with a higher concentration of d-galacturonic acid observed in the culture of this strain (Fig. 3).

## 4 Conclusions

Our results demonstrate that the relative contribution of the different sugar catabolic pathways to the overall physiology of *A. niger* during growth on plant biomass is not a direct reflection of the sugar composition of each substrate. WB and SBP are both abundant in pentoses and d-glucose, but SBP also contains significant amounts of d-galacturonic acid, d-galactose and l-rhamnose. However, significant growth reduction on these substrates was only observed for strain in which glycolysis, the PCP or all pathways were blocked. The strongest single pathway effect was observed for glycolysis, suggesting that when this pathway is blocked no other available components could provide *A. niger* with sufficient energy to maintain growth on plant biomass, likely due to CreA repression of alternative pathways due to d-glucose accumulation. The results of this study therefore exemplify the complexity of metabolic engineering to generate fungal cell factories for plant biomass based biochemicals. A deeper understanding of the interaction between metabolism and regulatory systems, and how this links to biomass degradation and sugar uptake is required to enable the design of efficient and robust fungal cell factories for a range of sugar-derived products.

## Funding sources

TC was supported by a grant of the NWO ALWOP.233 to RPdV. Part of the work was performed at the 10.13039/100019109Environmental Molecular Science Laboratory, a Department of Energy (DOE) Office of Science User Facility sponsored by the Office of Biological and Environmental Research (BER) and located at Pacific Northwest National Laboratory (PNNL). Pacific Northwest National Lab is operated by Battelle for the DOE under Contract DE-AC05-76RL01830. The 10.13039/501100002341Research Council of Finland grants no. 335246 and 348443, and the 10.13039/501100000329Novo Nordisk Foundation grant no. NNF21OC0067087 to MRM are also acknowledged.

## CRediT authorship contribution statement

**Tania Chroumpi:** Investigation, Formal analysis, Data curation, Visualization, Writing – original draft. **Astrid Müller:** Investigation, Formal analysis, Data curation, Visualization, Writing – original draft. **Mao Peng:** Formal analysis, Data curation, Visualization. **Mar Cubertorer Navarro:** Investigation. **Robin Kuijpers:** Investigation. **Agata Terebieniec:** Investigation. **Jiajia Li:** Formal analysis, Data curation, Visualization. **Lye Meng Markillie:** Formal analysis, Methodology, Data curation. **Hugh D. Mitchell:** Formal analysis, Methodology, Data curation. **Carrie D. Nicora:** Formal analysis, Methodology, Data curation. **Chelsea M. Hutchinson:** Formal analysis, Methodology, Data curation. **Vanessa Paurus:** Formal analysis, Methodology, Data curation. **Samuel O. Purvine:** Formal analysis, Methodology, Data curation. **Chaevien S. Clendinen:** Formal analysis, Methodology, Data curation. **Galya Orr:** Formal analysis, Methodology, Data curation. **Scott E. Baker:** Conceptualization, Supervision. **Miia R. Mäkelä:** Writing – review & editing. **Ronald P. de Vries:** Conceptualization, Funding acquisition, Supervision, Resources, Writing – review & editing.

## Declaration of competing interest

The authors declare the following financial interests/personal relationships which may be considered as potential competing interests:

Li Xu reports financial support was provided by Chinese Scholarship Council. If there are other authors, they declare that they have no known competing financial interests or personal relationships that could have appeared to influence the work reported in this paper.

## Data Availability

The RNAseq data of this study is available in the GEO database under accession number GSE196397 (https://www.ncbi.nlm.nih.gov/geo/query/acc.cgi?acc=GSE196397).

## Appendix Supplementary materials

Image, application 1

Image, application 2

Image, application 3
