# Supplementary material for: Metabolic engineering reveals the relative importance of different sugar catabolic pathways during consumption of plant biomass by Aspergillus niger
Source: Curr Res Microb Sci. 2025 Aug 5;9:100454. doi: 10.1016/j.crmicr.2025.100454 (PMC12744346; doi:10.1016/j.crmicr.2025.100454)
Supplement: Supplementary file 3 [file mmc3.pdf]

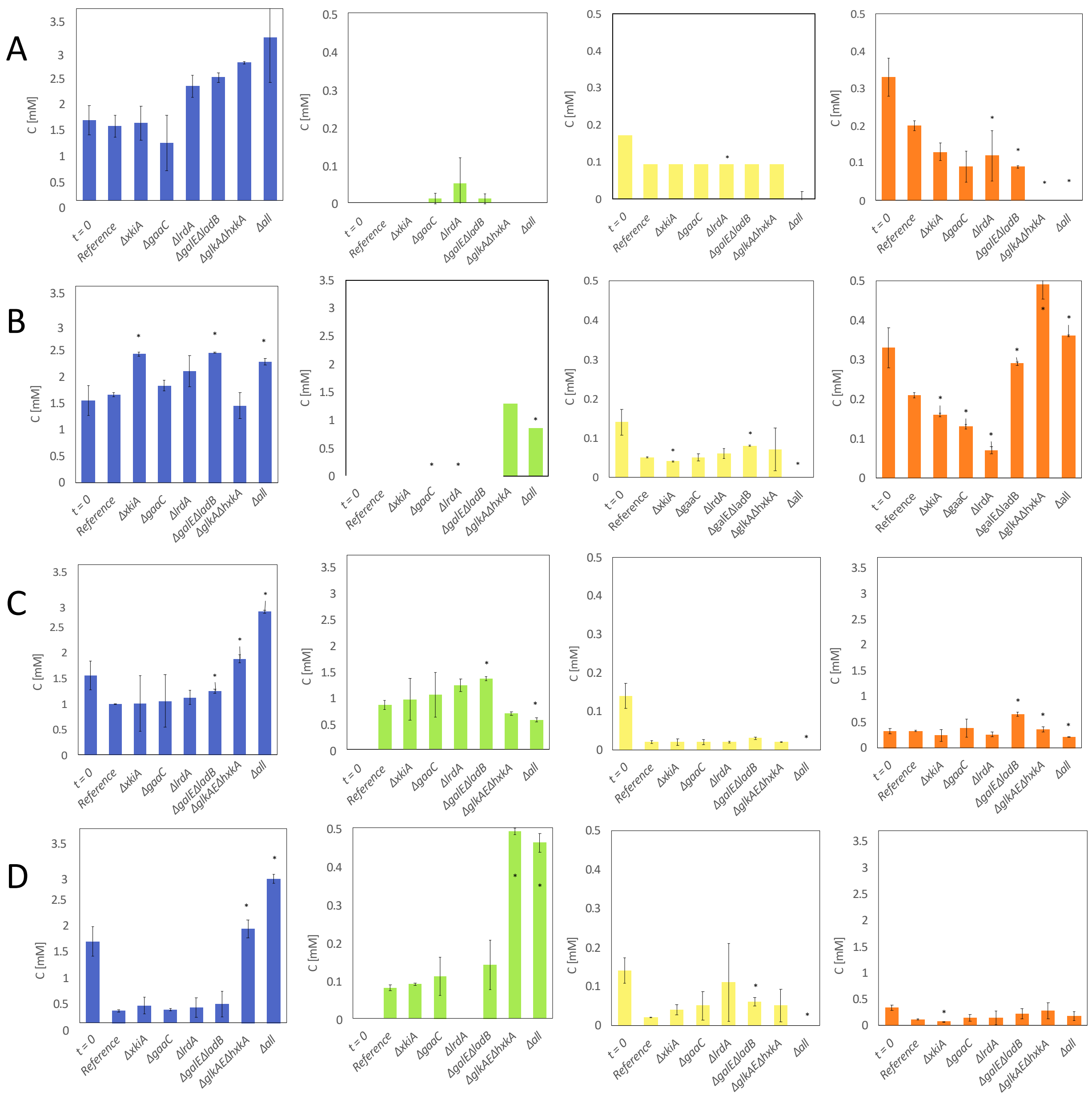

Figure S1A. Sugar analysis of the *A. niger* reference strain and deletion strains on 1% wheat bran. Concentrations of D-glucose (blue), D-xylose (green), D-galactose (yellow), L- arabinose (orange) were measured after 2 (**A**), 4 (**B**), 8 (**C**), 24 hours (**D**). Analysis was carried out using biological triplicates. Statistical significance is represented by * ($p < 0.05$). t = 0 indicates the sugars present in the medium before the strain was added.

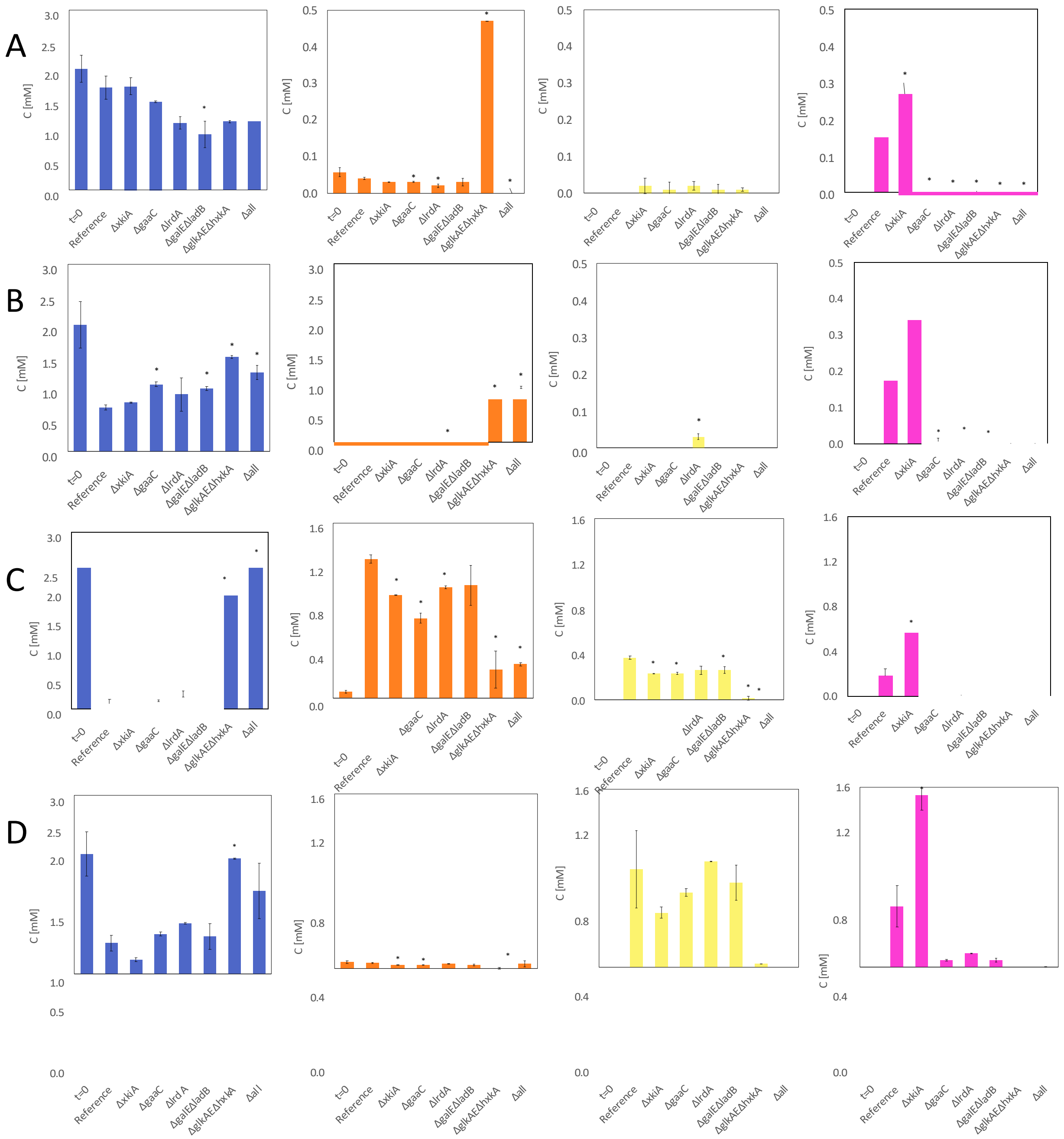

Figure S1B. Sugar analysis of the *A. niger* reference strain and deletion strains on 1% sugar beet pulp. Concentrations of D-glucose (blue), L-arabinose (orange), D-galactose (yellow), Galacturonic acid (pink) were measured after 2 (**A**), 4 (**B**), 8 (**C**), 24 hours (**D**). Analysis was carried out using biological triplicates. Statistical significance is represented by * ($p < 0.05$). t = 0 indicates the sugars present in the medium before the strain was added.

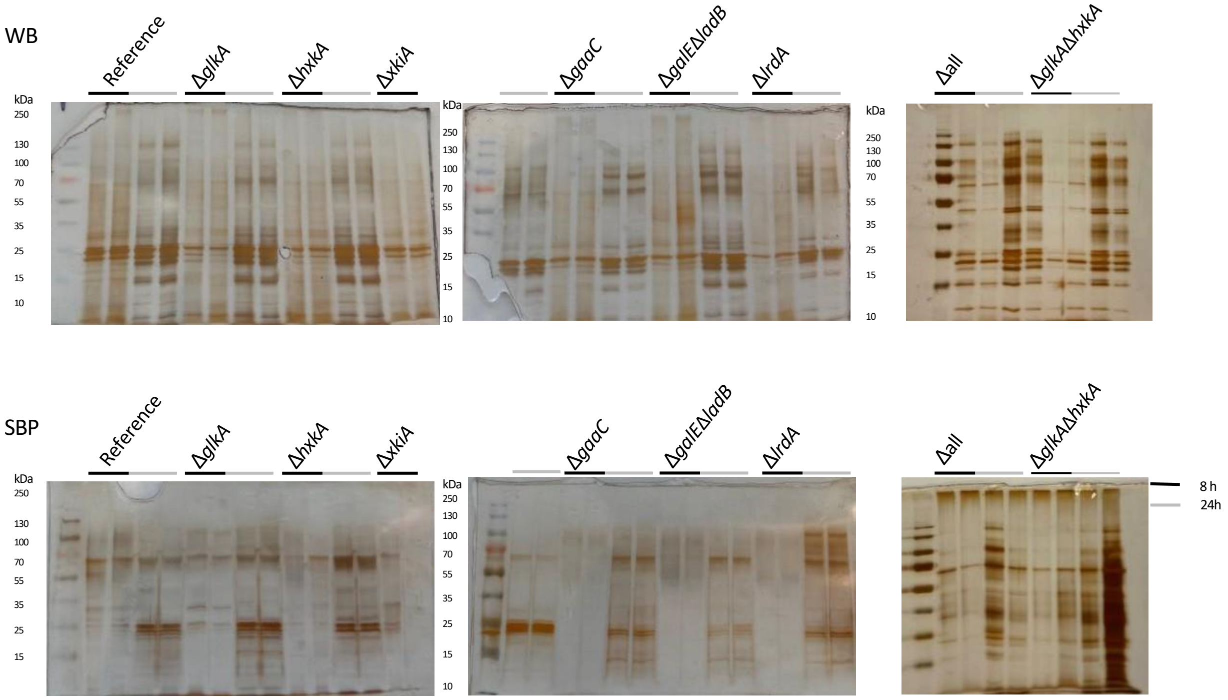

Figure S 2. SDS-PAGE analysis of the extracellular protein profile of the *A. niger* reference strains and catabolic pathway mutants after 8 and 24 h incubation with 1% wheat bran (WB) and 1% sugar beet pulp (SBP).

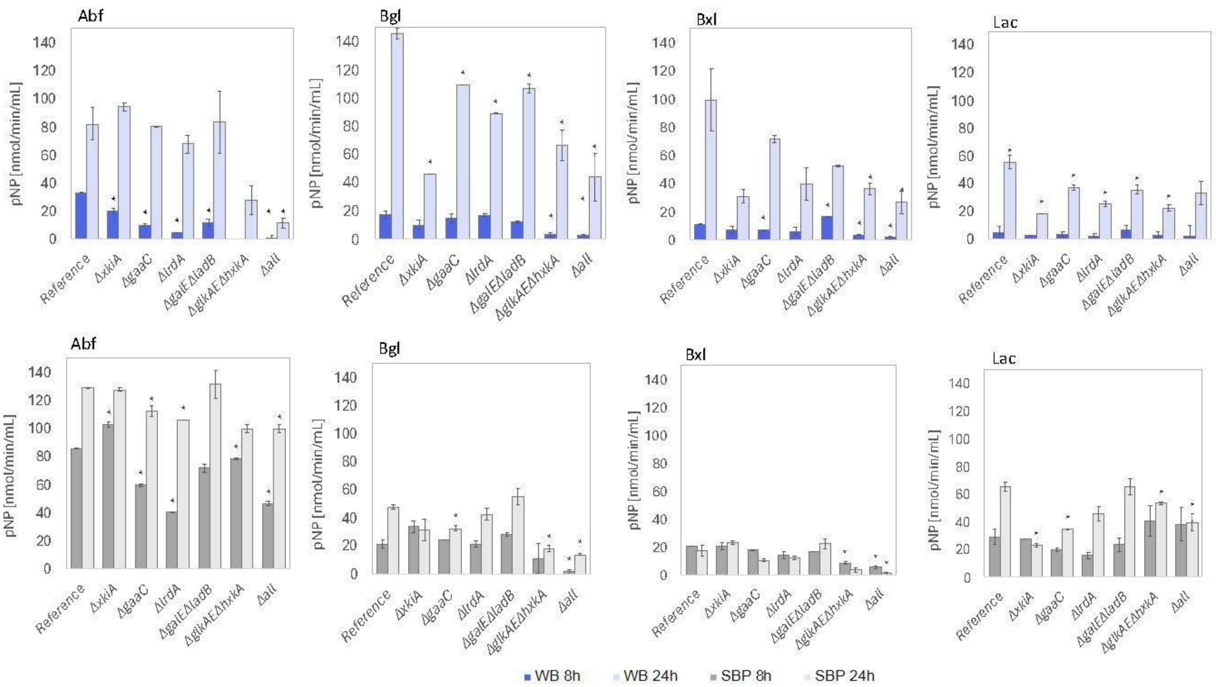

Figure S3. Extracellular enzyme activities of selected enzymes involved in plant biomass degradation produced by the *A. niger* reference strains and catabolic pathway mutants after 8 and 24 h incubation with 1% wheat bran (WB) and 1% sugar beet pulp (SBP). Abf = α-L-arabinofuranosidase, Bgl = β-D-glucosidase, Bxl = β-D-xylosidase, Lac = β-D-galactosidase.

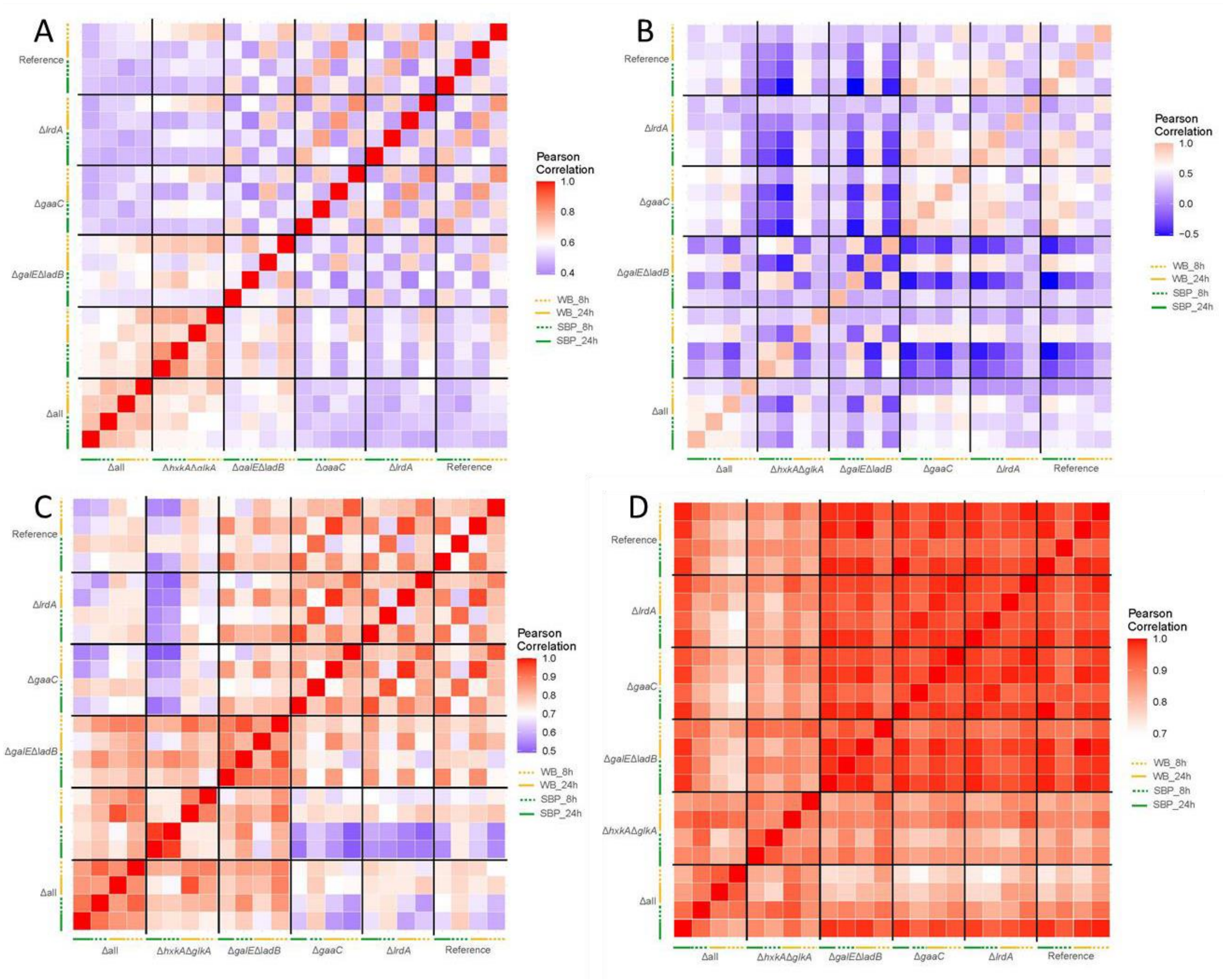

Figure S4. Correlation matrix of the proteome and metabolome of all tested strains. A: Intracellular proteome, B: Extracellular proteome, C: Intracellular metabolome, D: Extracellular metabolome.

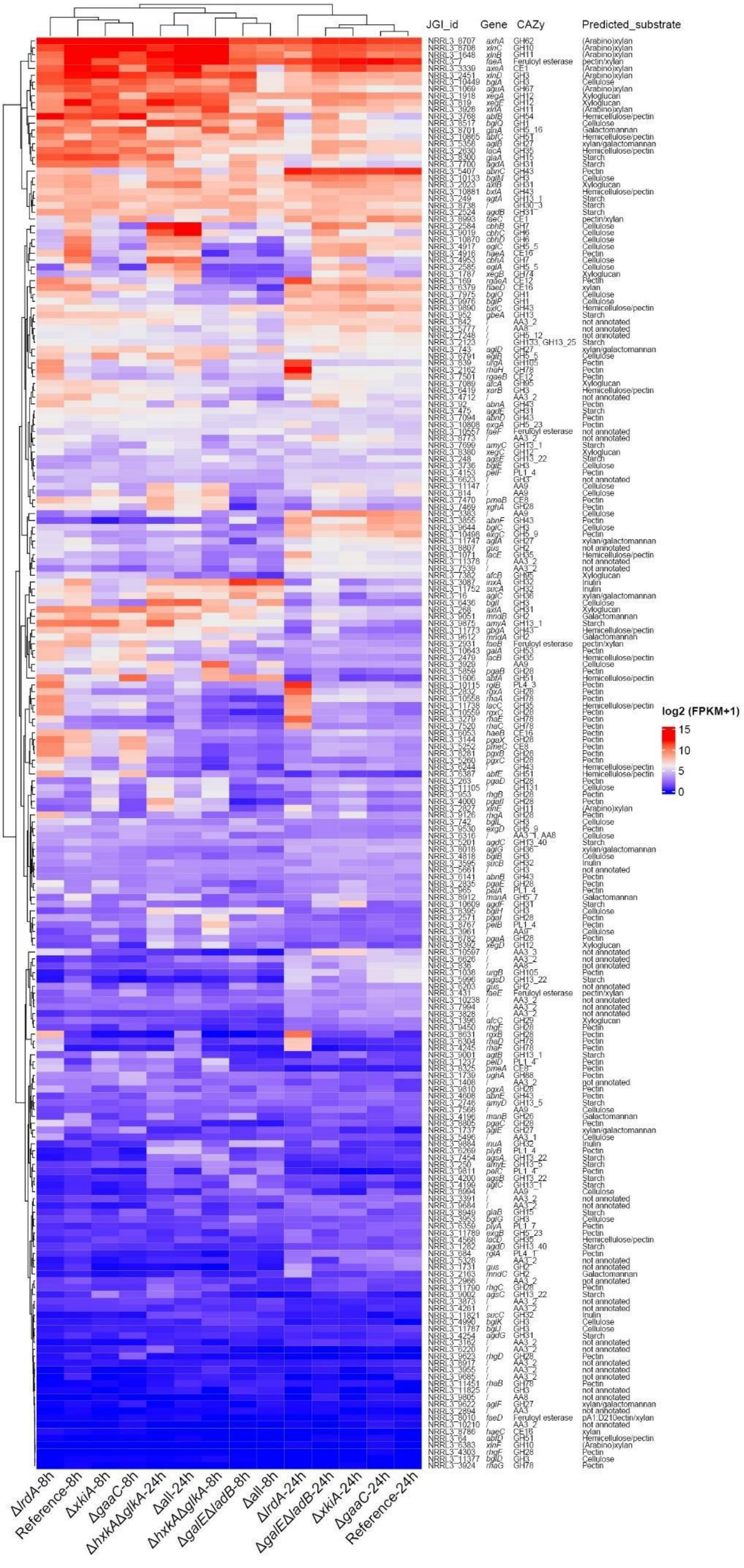

Figure S5A. Transcriptome analysis of CAZy genes encoding plant biomass degrading enzymes in the reference and metabolic mutants during growth on wheat bran. Samples were analyzed after 8 and 24 h. The gene id from the *A. niger* NRRL3 genome (https://mycocosm.jgi.doe.gov/Aspni_NRRL3_1/Aspni_NRRL3_1.home.html), known gene names, the CAZy family they belong to and the polysaccharide they act on are indicated.

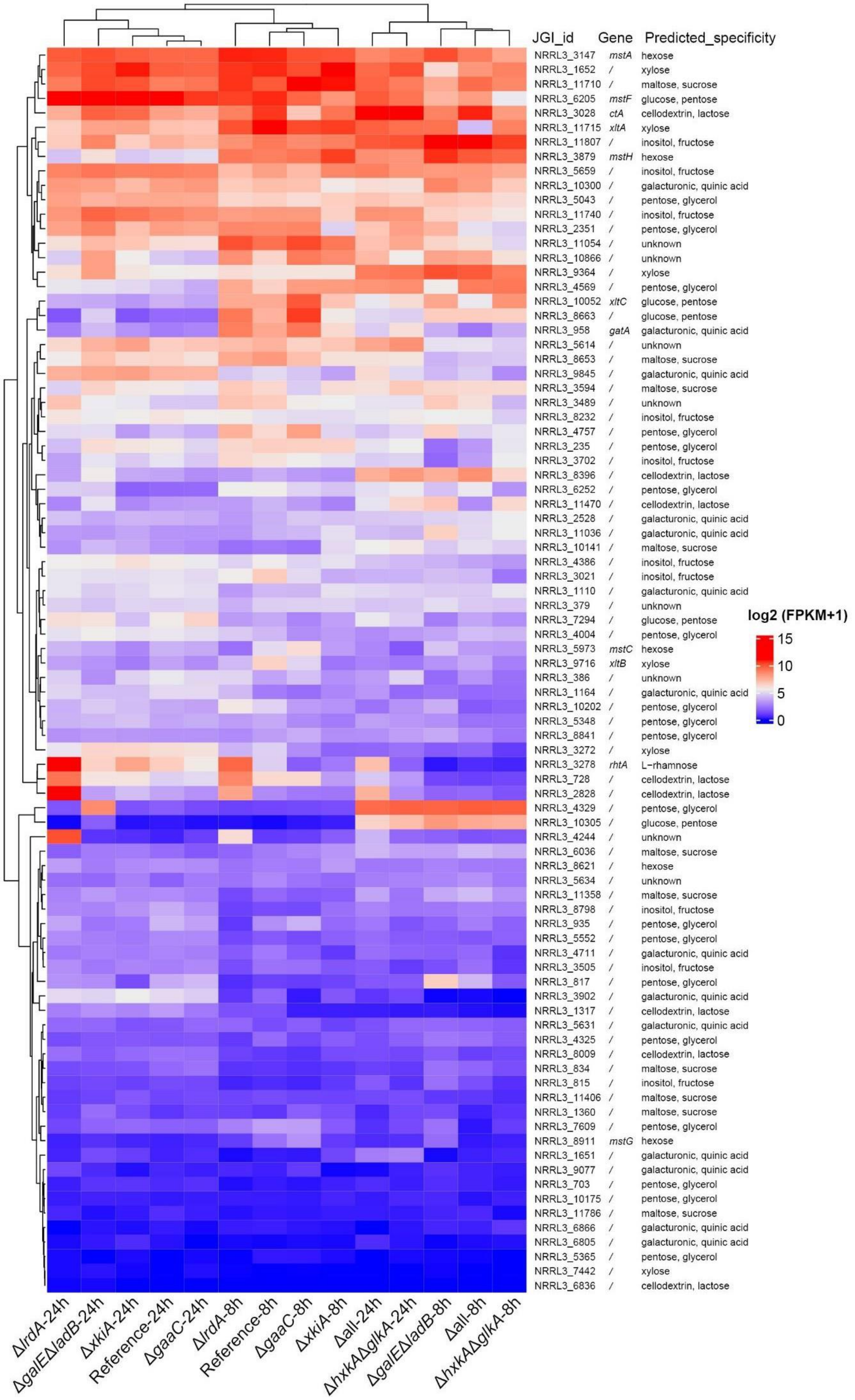

Figure S5B. Transcriptome analysis of genes encoding sugar transporters in the reference and metabolic mutants during growth on wheat bran. Samples were analyzed after 8 and 24 h. The gene id from the *A. niger* NRRL3 genome (https://mycocosm.jgi.doe.gov/Aspni_NRRL3_1/Aspni_NRRL3_1.home.html), known gene names, and the putative sugar they transport are indicated.

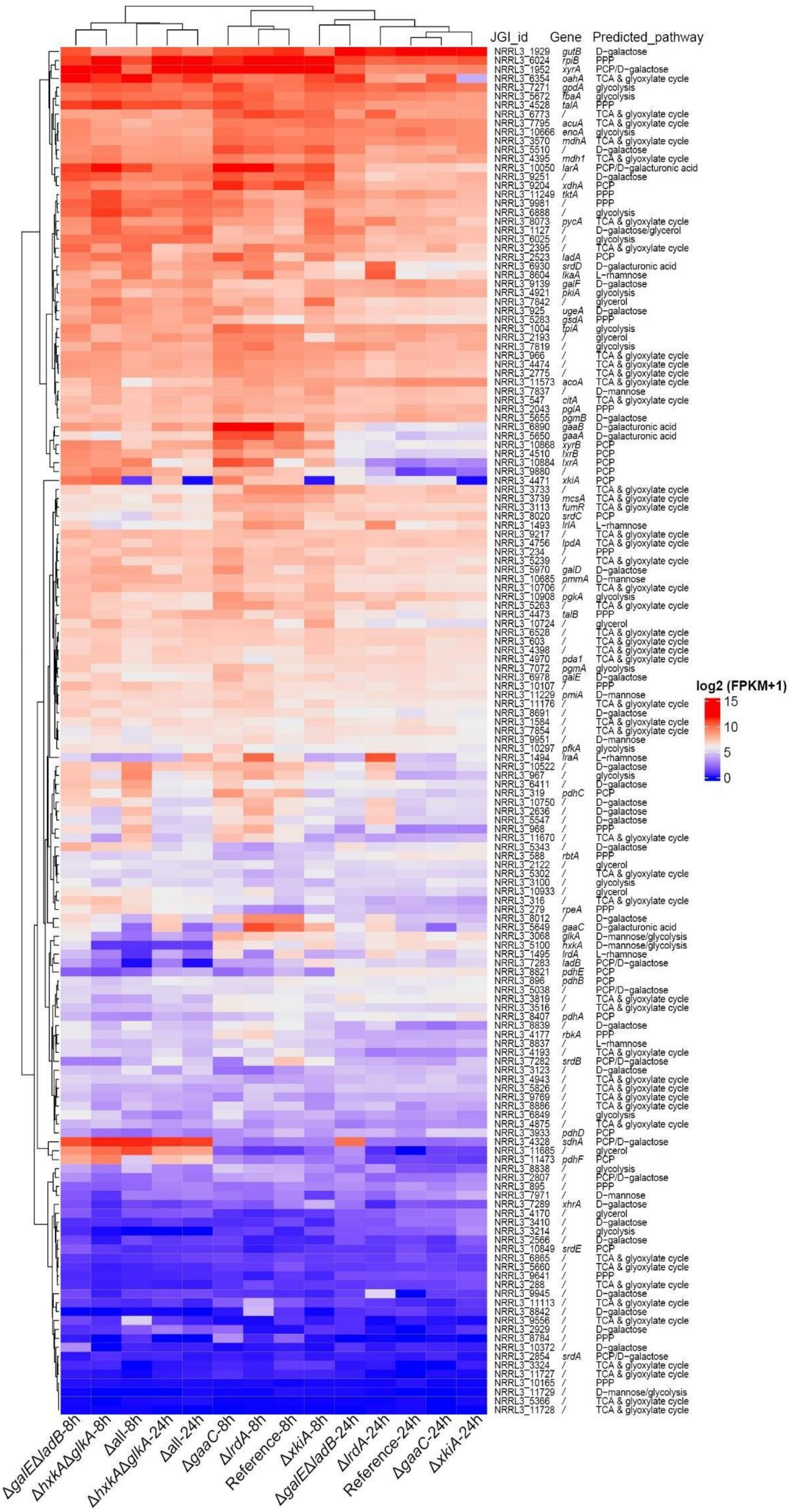

Figure S5C. Transcriptome analysis of genes encoding sugar metabolic genes in the reference and metabolic mutants during growth on wheat bran. Samples were analyzed after 8 and 24 h. The gene id from the *A. niger* NRRL3 genome (https://mycocosm.jgi.doe.gov/Aspni_NRRL3_1/Aspni_NRRL3_1.home.html), known gene names, and the pathway they belong to are indicated.

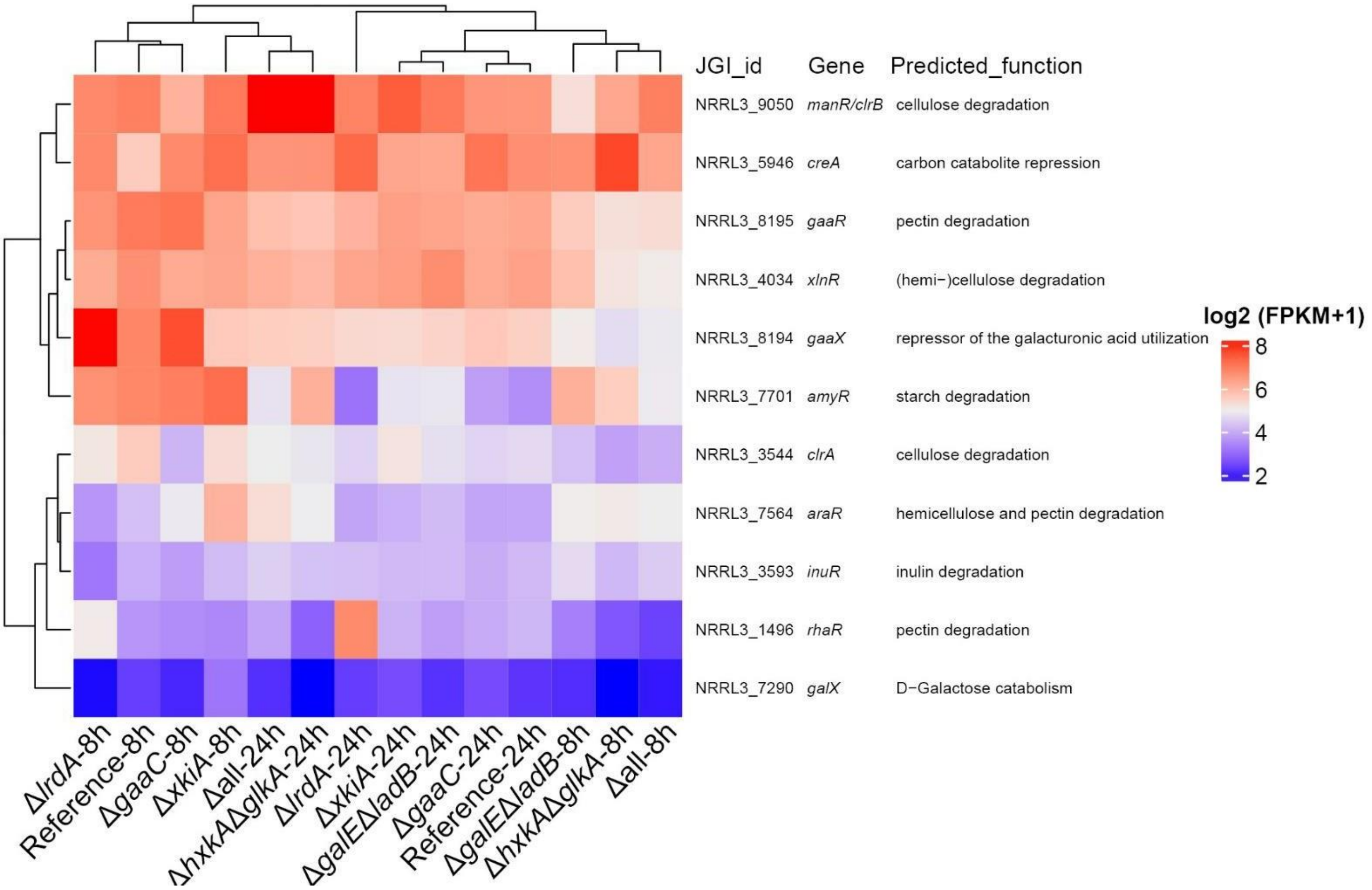

Figure S5D. Transcriptome analysis of genes encoding plant biomass conversion related transcriptional regulators in the reference and metabolic mutants during growth on wheat bran. Samples were analyzed after 8 and 24 h. The gene id from the *A. niger* NRRL3 genome (https://mycocosm.jgi.doe.gov/Aspni_NRRL3_1/Aspni_NRRL3_1.home.html), gene names, and the process they regulate are indicated.

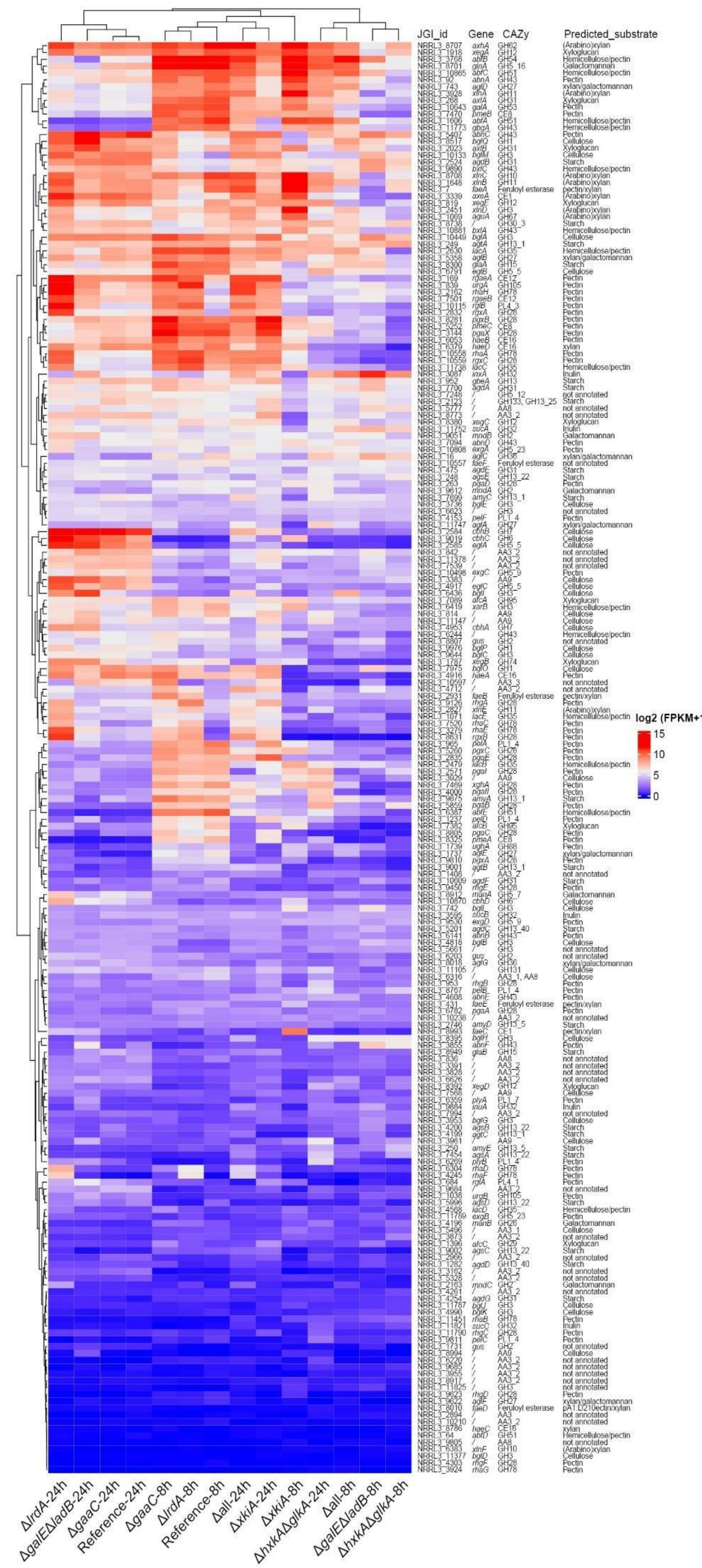

Figure S5E. Transcriptome analysis of CAZy genes encoding plant biomass degrading enzymes in the reference and metabolic mutants during growth on sugar beet pulp. Samples were analyzed after 8 and 24 h. The gene id from the *A. niger* NRRL3 genome (https://mycocosm.jgi.doe.gov/Aspni_NRRL3_1/Aspni_NRRL3_1.home.html), known gene names, the CAZy family they belong to and the polysaccharide they act on are indicated.

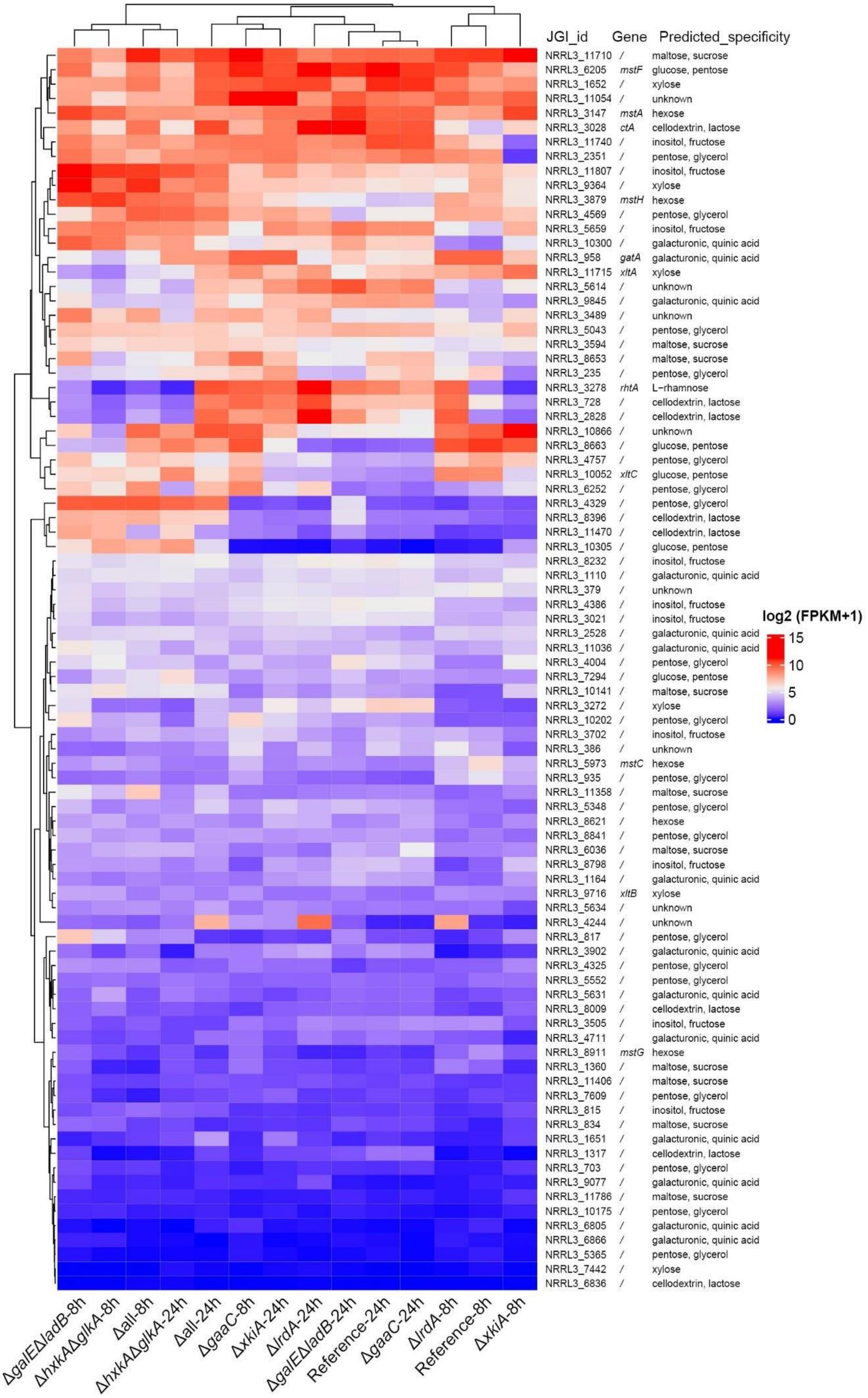

Figure S5F. Transcriptome analysis of genes encoding sugar transporters in the reference and metabolic mutants during growth on sugar beet pulp. Samples were analyzed after 8 and 24 h. The gene id from the *A. niger* NRRL3 genome (https://mycocosm.jgi.doe.gov/Aspni_NRRL3_1/Aspni_NRRL3_1.home.html), known gene names, and the putative sugar they transport are indicated.

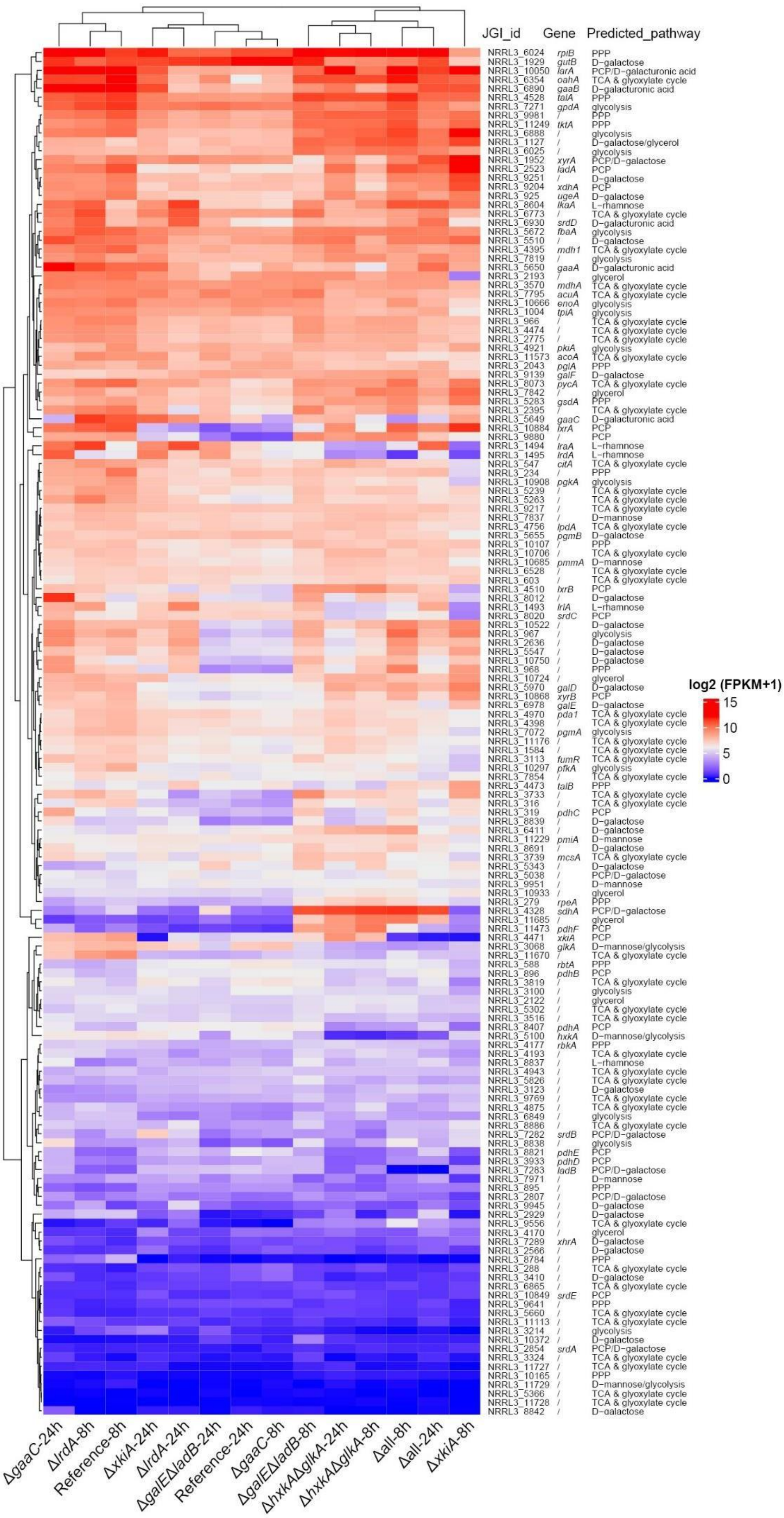

Figure S5G. Transcriptome analysis of genes encoding sugar metabolic genes in the reference and metabolic mutants during growth on sugar beet pulp. Samples were analyzed after 8 and 24 h. The gene id from the *A. niger* NRRL3 genome (https://mycocosm.jgi.doe.gov/Aspni_NRRL3_1/Aspni_NRRL3_1.home.html), known gene names, and the pathway they belong to are indicated.

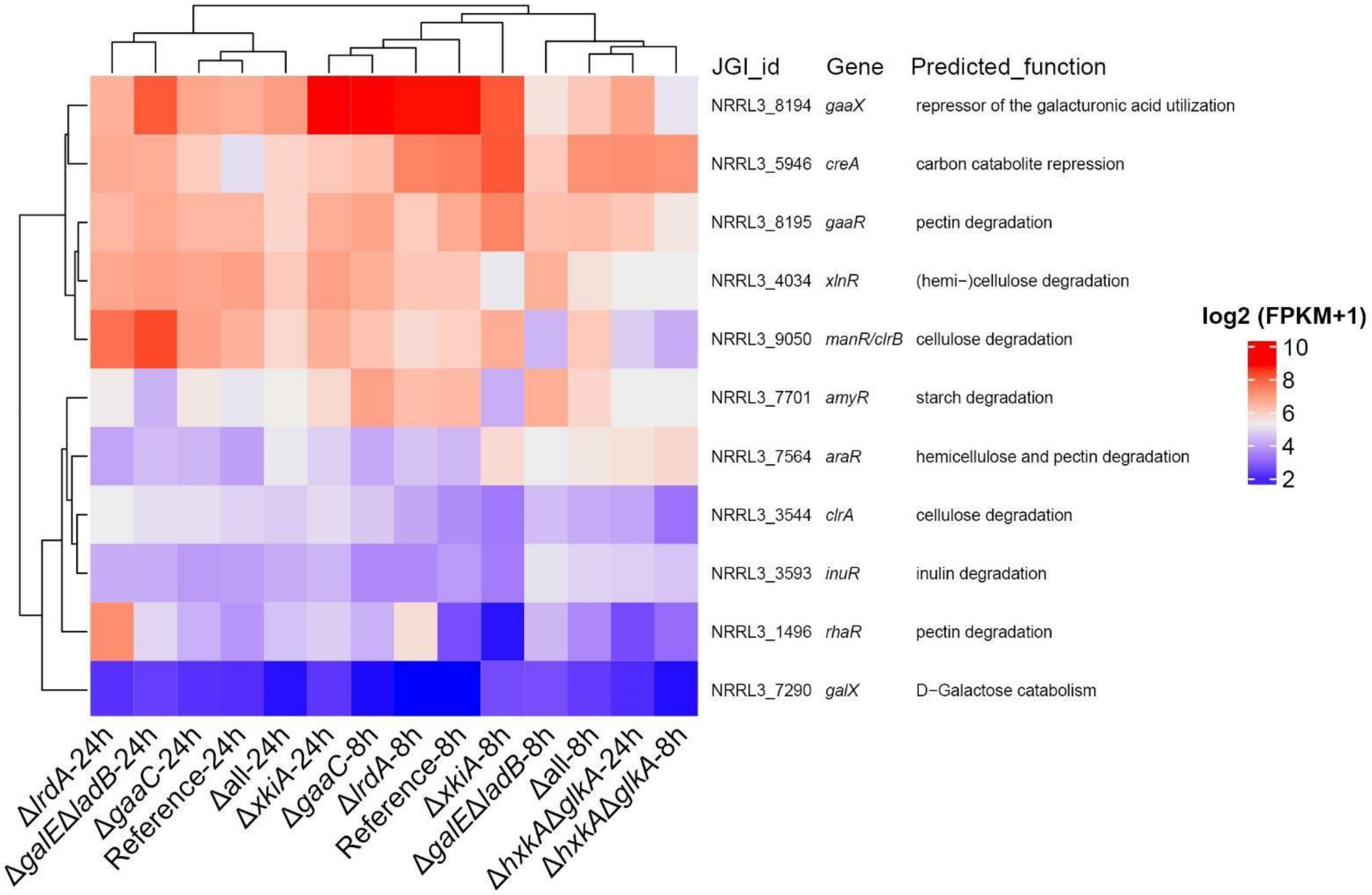

Figure S5H. Transcriptome analysis of genes encoding plant biomass conversion related transcriptional regulators in the reference and metabolic mutants during growth on sugar beet pulp. Samples were analyzed after 8 and 24 h. The gene id from the *A. niger* NRRL3 genome (https://mycocosm.jgi.doe.gov/Aspni_NRRL3_1/Aspni_NRRL3_1.home.html), gene names, and the process they regulate are indicated.

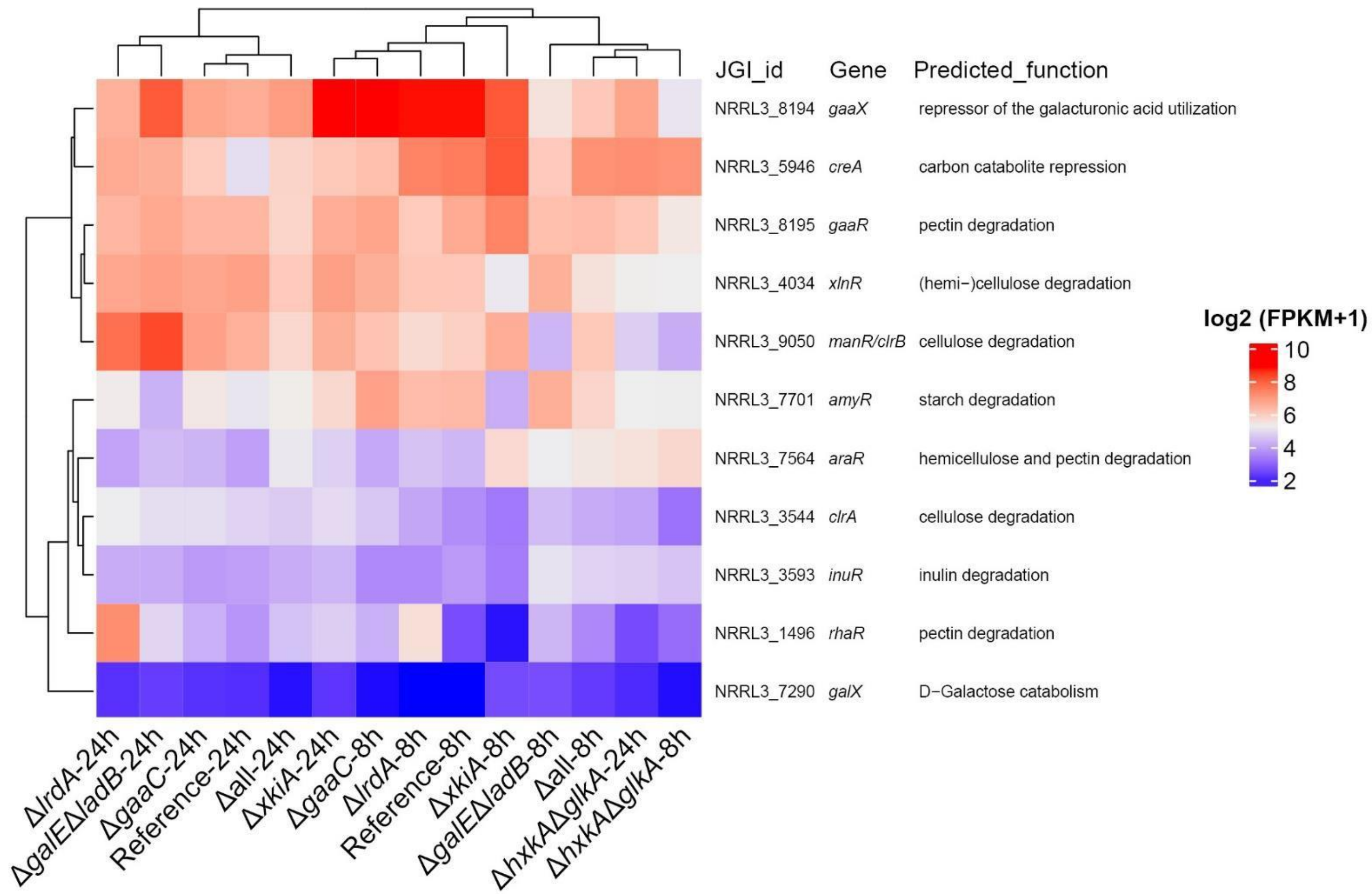

Figure S5H. Transcriptome analysis of genes encoding plant biomass conversion related transcriptional regulators in the reference and metabolic mutants during growth on sugar beet pulp. Samples were analyzed after 8 and 24 h. The gene id from the *A. niger* NRRL3 genome (https://mycocosm.jgi.doe.gov/Aspni_NRRL3_1/Aspni_NRRL3_1.home.html), gene names, and the process they regulate are indicated.
